# Urbanization and physical activity in the global Prospective Urban and Rural Epidemiology study

**DOI:** 10.1038/s41598-022-26406-5

**Published:** 2023-01-06

**Authors:** Kwadwo Boakye, Marit Bovbjerg, John Schuna, Adam Branscum, Ravi Prasad Varma, Rosnah Ismail, Olga Barbarash, Juan Dominguez, Yuksel Altuntas, Ranjit Mohan Anjana, Rita Yusuf, Roya Kelishadi, Patricio Lopez-Jaramillo, Romaina Iqbal, Pamela Serón, Annika Rosengren, Paul Poirier, P. V. M. Lakshmi, Rasha Khatib, Katarzyna Zatonska, Bo Hu, Lu Yin, Chuangshi Wang, Karen Yeates, Jephat Chifamba, Khalid F Alhabib, Álvaro Avezum, Antonio Dans, Scott A Lear, Salim Yusuf, Perry Hystad

**Affiliations:** 1 Department of Public Health and Health Services Administration, California State University, Chico, CA USA; 2 College of Public Health and Human Sciences, Oregon State University, 2520 SW Campus Way, Corvallis, OR 97331 USA; 3 Achutha Menon Centre for Health Science Studies, Sree Chitra Tirunal Institute for Medical Sciences and Technology, Trivandrum, India; 4 Health Action By People, Thiruvananthapuram, India; 5 Department of Community Health, Faculty of Medicine, University Kebangsaan Malaysia, Medical Center, Kuala Lumpur, Malaysia; 6 Federal State Budgetary Institution Research Institute for Complex Issues of Cardiovascular Diseases, Kemerovo, Russian Federation; 7 Estudios Clínicos Latino América, 160 Rosario, Argentina; 8 Instituto Cardiovascular de Rosario, Oroño 450, Rosario, Argentina; 9 Department of Endocrinology and Metabolism, University of Health Sciences, Sisli Hamidiye Etfal Teaching and Research Hospital, Istanbul, Turkey; 10 Madras Diabetes Research Foundation, Chennai, India; 11 School of Life Sciences, Independent University, Dhaka, Bangladesh; 12 Isfahan Cardiovascular Research Center, Cardiovascular Research Institute, Isfahan University of Medical Sciences, Isfahan, Iran; 13 Masira Research Institute, Medical School, Universidad de Santander (UDES), Bucaramanga, Colombia; 14 Department of Community Health Sciences and Medicine, Aga Khan University, Stadium Road, Karachi, Pakistan; 15 Faculty of Medicine, Universidad de La Frontera, Claro Solar 115, Temuco, Chile; 16 Sahlgrenska University Hospital, Göteborg, Sweden; 17 Department of Molecular and Clinical Medicine, Sahlgrenska Academy, University of Gothenburg, Göteborg, Sweden; 18 Institut Universitaire de Cardiologie et de Pneumologie de Quebec, Québec, Canada; 19 Department of Community Medicine & School of Public Health, Postgraduate Institute of Medical Education and Research (PGIMER), Chandigarh, India; 20 Advocate Aurora Research Institute, Advocate Aurora Health, Downers Grove, IL USA; 21 Institute of Community and Public Health, Birzeit University, Birzeit, Palestine; 22 Department of Social Medicine, Wroclaw Medical University, Wroclaw, Poland; 23 Fuwai Hospital, Chinese Academy of Medical Sciences, National Center for Cardiovascular Diseases, Beijing, China; 24 Department of Medicine, Queen’s University, Kingston, ON Canada; 25 Physiology Department, College of Health Sciences, University of Zimbabwe, Harare, Zimbabwe; 26 Department of Cardiac Sciences, King Fahad Cardiac Center, College of Medicine, King Saud Medical City, King Saud University, Riyadh, Saudi Arabia; 27 International Research Center, Hospital Alemão Oswaldo Cruz, Avenida Paulista, São Paulo, Brazil; 28 Department of Medicine, University of the Philippines, Manila, Philippines; 29 Faculty of Health Sciences, Simon Fraser University, Burnaby, BC Canada; 30 Population Health Research Institute, McMaster University and Hamilton Health Sciences, Hamilton, ON Canada

**Keywords:** Environmental impact, Socioeconomic scenarios, Epidemiology, Lifestyle modification

## Abstract

Urbanization may influence physical activity (PA) levels, although little evidence is available for low- and middle- income countries where urbanization is occurring fastest. We evaluated associations between urbanization and total PA, as well as work-, leisure-, home-, and transport-specific PA, for 138,206 adults living in 698 communities across 22 countries within the Prospective Urban and Rural Epidemiology (PURE) study. The 1-week long-form International PA Questionnaire was administered at baseline (2003–2015). We used satellite-derived population density and impervious surface area estimates to quantify baseline urbanization levels for study communities, as well as change measures for 5- and 10-years prior to PA surveys. We used generalized linear mixed effects models to examine associations between urbanization measures and PA levels, controlling for individual, household and community factors. Higher community baseline levels of population density (− 12.4% per IQR, 95% CI − 16.0, − 8.7) and impervious surface area (− 29.2% per IQR, 95% CI − 37.5, − 19.7), as well as the rate of change in 5-year population density (− 17.2% per IQR, 95% CI − 25.7, − 7.7), were associated with lower total PA levels. Important differences in the associations between urbanization and PA were observed between PA domains, country-income levels, urban/rural status, and sex. These findings provide new information on the complex associations between urbanization and PA.

## Introduction

Urbanization can be broadly defined as the process by which large numbers of people become permanently concentrated in small areas, which results in changes to economic, social and environmental factors^1^. Approximately 55% of the world’s population now lives in urban areas, which is expected to increase to 68% (approximately 7 billion people) by 2050^2^. Most of this increase, up to 90%, will occur in Asia and Africa^2^. Trends in urbanization are altering the landscape of human settlement, with significant implications for living conditions, lifestyles, work conditions, and the environment in different parts of the world^3^. In particular, urbanization may influence the amount and type of individual physical activity (PA) levels^4,5^.

PA is one of the most important components of successful health promotion and disease prevention^6–8^. Physical inactivity causes more than 3 million deaths per year, of which 2.6 million occur in low- and middle-income countries (LMICs)^9^. Despite the health benefits associated with being physically active^10^, levels of PA among adults have been decreasing over the last several decades^11^. Globally, only 1 in 4 adults achieve current WHO guidelines for PA (at least 150–300 min of moderate-intensity aerobic PA per week)^12–14^. This ranges from a high of 39% in the Americas to a low of 22% in African regions^11^. The global variability in urbanization and PA has been examined at an ecological level^15–18^, but few studies have used individual-level data to examine the association between urbanization levels and PA, especially in LMICs^4,5,19^.

Urbanization is likely to influence types of PA (PA domains) in differing ways. Major PA domains include transport, leisure/recreational, household, and occupational^20,21^, and the proportion of PA from each domain varies greatly between countries and geographic regions^22^. For example, PA in LMICs is most often accumulated in occupational, household, and transport domains, whereas leisure-time PA contributes more to total PA in high-income countries (HICs)^22^. Given the potential effect of urbanization as an upstream driving factor of PA^23,24^, there is surprisingly little research that examines the association between urbanization and individual components of PA in LMICs^4,5,18^, countries where the most rapid urbanization is currently occuring^25,26^. The few studies that have been done in HICs have mostly evaluated the influence of urbanization on leisure/recreational and transport PA, with conflicting results^5,27^.

There are many pathways through which urbanization could be associated with individual PA levels. Urbanization results in substantial transformations to economic activity, socioeconomic conditions, occupational activities, and social, cultural and physical environments^1^. These structural changes lead to individual lifestyle changes that can directly and indirectly influence PA levels^28^. For example, urbanization can result in dramatic changes to the occupational landscape^29^, typically increasing white collar and service jobs. This results in a transition away from labor intensive jobs to those that are more sedentary, resulting in less occupational PA. Similarly, urbanization may lead to technological advances that result in an increase in household technology such as microwaves and washing machines^28^. Such technological changes may reduce the physical demands of home activities and result in a decline of household PA. Urbanization also brings changes in terms of the built environment and how it promotes or inhibits individuals recreation and transportation PA^30–32^. Urbanization generally leads to an increase in ownership and use of motorized vehicles, resulting in a decline of active transport PA^33,34^. While the rate of urbanization change and its consequences on overall health are well documented^1,35^, few studies have specifically examined the independent effect of urbanization on PA levels of adults, especially in LMICs undergoing rapid urbanization^25,26^.

The objective of this research is to determine how urbanization is associated with total and domain specific PA across 698 diverse communities in 22 low-, middle-, and high-income countries using individual data on 138,206 adults in the Prospective Urban Rural Epidemiology (PURE) study. This analysis provides new information on how urbanization is associated with total and domain specific PA, and how these relationships vary between country-income status and by individual socio-demographic characteristics. Understanding how PA is related to urbanization is central to reducing population level physical inactivity, especially among rapidly developing and urbanizing LMICs.

## Results

We restricted our analyses to the 138,206 PURE participants who had complete PA and urbanization measures (Table 1). The mean age of all participants was 50.5 years, 58.7% of participants were females, 50.2% lived in urban areas, and 11.8% of participants were from HICs, 75.0% from MICs and 13.2% from LICs. The mean total PA level was 4140 MET minutes per week (sd = 4984), varying from 4480 MET minutes per week for the lowest quartile (Q1) of population density change to 3825 MET minutes per week for the highest quartile (Q4) of population density change (Table 2). Household and occupational PA contributed the most to overall PA levels.

**Table 1:** Characteristics of 138,206 PURE participants residing in 698 communities across 22 countries that are included in the analyses of urbanization and physical activity.

Characteristic	
Participants, n	138,206
Age, mean (SD)	50.5 (9.8)
**Sex**	
Female, n (%)	81,051 (58.7)
Male, n (%)	57,155 (41.3)
**Education**	
None, primary, or unknown, n (%)	55,809 (40.4)
Secondary, n (%)	54,019 (39.1)
Trade, college, or university, n (%)	28,128 (20.4)
**Household wealth index**	
Low, n (%)	42,181 (30.5)
Middle, n (%)	46,784 (33.9)
High, n (%)	47,665 (34.5)
**BMI**	
< 20, n (%)	10,572 (7.7)
20–30, n (%)	94,936 (68.7)
> 30, n (%)	24,589 (17.8)
Missing, n (%)	8109 (5.9)
**Chronic conditions at baseline**	
No, n (%)	110,674 (80.1)
Yes, n (%)	27,532 (19.9)
**Urban/rural status**	
Urban, n (%)	69,347 (50.2)
Rural, n (%)	68,859 (49.8)
**Country income level**	
High, n (%)	16,302 (11.8)
Middle, n (%)	103,666 (75.0)
Low, n (%)	18,238 (13.2)

**Table 2:** Participant physical activity levels stratified by quartiles of 5-year community population density change.

	All participants	5-year community population density change			
		Quartile 1 (least change)	Quartile 2	Quartile 3	Quartile 4 (most change)
No. individuals	138,206	36,742	33,170	34,240	34,054
Total PA, MET × min per week, mean (SD)	4140 (4984)	4480 (5035)	4259 (4883)	3975 (4820)	3825 (5156)
Transport PA	629 (1237)	597 (1210)	640 (1194)	689 (1259)	596 (1281)
Recreational PA	511 (1140)	453(1046)	573 (1123)	552 (1118)	472 (1263)
Household PA	1469 (2367)	1613 (2286)	1317 (2195)	1509 (2465)	1424 (2502)
Occupational PA	1533 (3983)	1819 (4169)	1729 (3997)	1229 (3650)	1338 (4049)

### Distribution of urbanization metrics

**Figure 1:**
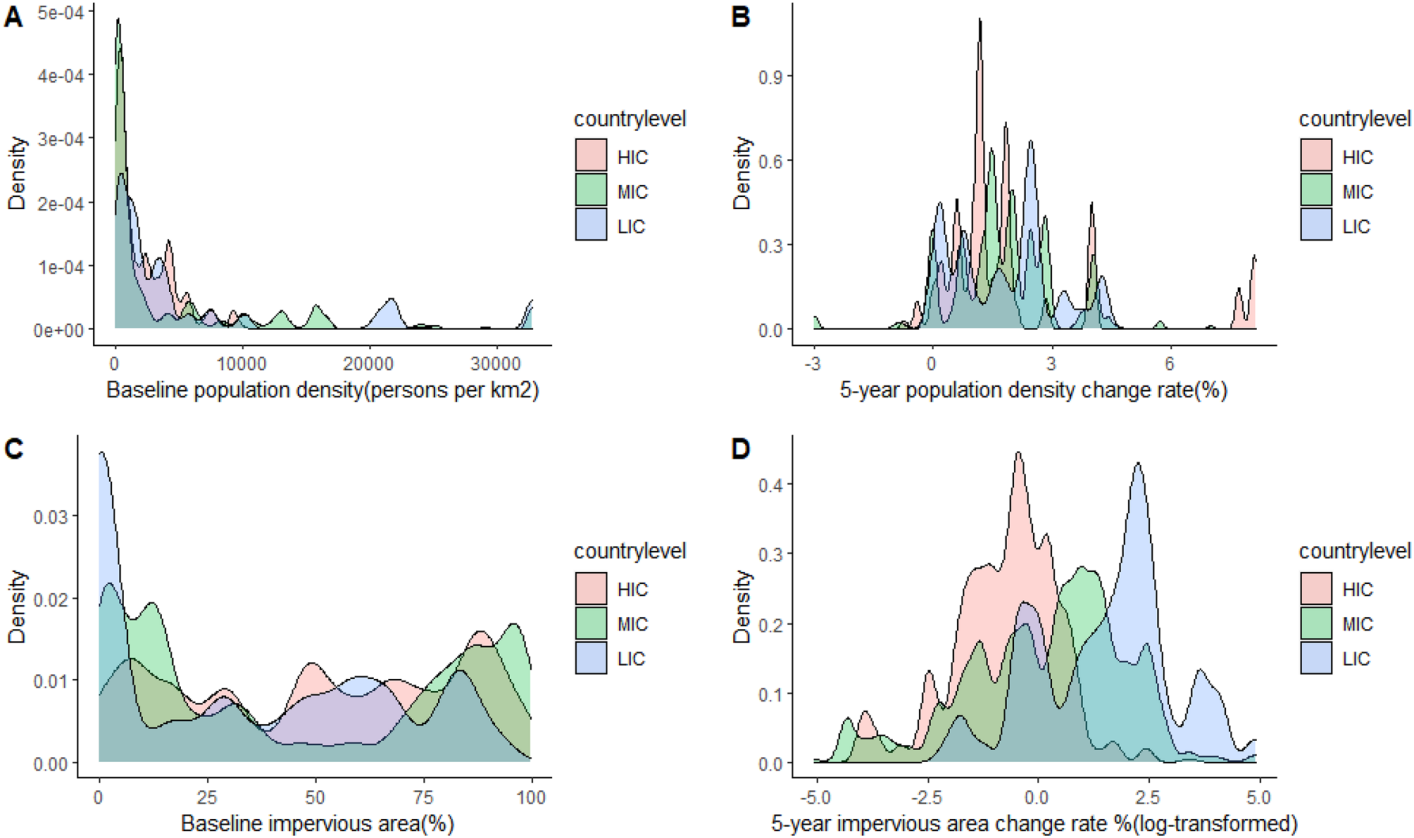
Baseline and 5-year change measures of community population density and impervious surface area for 138,206 PURE participants.

The distribution of urbanization metrics for PURE participants is shown in Fig. 1, stratified by country income level. Due to outliers and the highly right-skewed distribution for impervious area change we present log-transformed measures. The baseline median (IQR) population density and percent impervious area for PURE communities was 802 people/sq km (4428) and 35% (73) respectively. Over a 5-year period prior to baseline, the median (IQR) annual population density and impervious surface area change rate were 2% (1.5) and 0.4% (2) per year, respectively. For each urbanization measure, we observed a range of baseline levels and change rates by HIC, MIC and LICs, indicating that urbanization is occurring in all country income classes but is greatest in LMICs. There were varying levels of correlation between our different urbanization metrics and spatial and temporal scales of analyses (Appendix, Table S1. For different buffer areas around community centroids (1 and 5 km’s), and for different timescales of change (5 and 10 years) there were high correlations for all metrics (all r > 0.89 and > 0.91, respectively). For this reason, we only present 1 km and 5-year urbanization metrics below.

### Associations between population density and physical activity

Table 3 summarizes models examining baseline population density and change in population density for total and domain specific PA. In adjusted models, an IQR increase in baseline population density was associated with a − 12.4% (95% CI − 16.0, − 8.7) lower population median of MET-min of total PA, while an IQR increase in the 5-year change in population density was associated with a − 17.2% (95% CI − 25.7, − 7.7) lower population median of total PA. The largest negative associations were observed for occupational and household PA, while positive associations were observed for transport and recreational PA. Statistically significant differences in the association of baseline population density on total PA, transport PA, recreation PA and occupation PA were observed for each of HIC, MIC and LIC. For change in population density, statistically significant differences by country-income class was observed for household PA. Larger magnitudes of associations were typically observed for HICs, compared to MIC or LICs, expect for population density change and recreational PA.

**Table 3:** Associations between community population density at baseline, and 5-year population density change prior to baseline, and individual-level physical activity.

Population density at baseline (β per IQR = 4428 people/sq. km)						5-year change in population density (β per IQR = 2% increase per year)				
	All	HIC	MIC	LIC	*p* value^†^	All	HIC	MIC	LIC	*p* value^†^
Total PA	− 12.4 (− 6.0, − .7)	− 6.2 (− 6.5, − .6)	− .3 (− 1.4, 3.3)	− 6.2 (− 0.2, − 2.4)	0.04	− 7.2 (− 5.7, − .7)	− 5.7 (− 0.6, − 0.2)	− 3.3 (− 4.8, − .1)	− 2.6 (− 8.5, − .4)	0.17
Transport	0.8 (− .7,6.6)	103.9 (65.6, 151.2)	29.3 (17.5, 42.2)	− 6.2 (− 0.9, − 1.3)	< 0.001	7 (− .0, 23.0)	− 1.6 (− 4.5, − 5.6)	13.8 (− .4, 35.4)	16.2 (− 5.8, 9.3)	0.30
Recreation	9.3 (3.7, 15.1)	6.4 (− 2.9, 29.9)	36.9 (24.5, 50.7)	− .3 (− .4, 0.2)	< 0.001	6.3 (− .1, 21.6)	6.4 (− 6.8, 36.1)	11.6 (− .2, 32.8)	− 2.2 (− 6.1, − .2)	0.53
Household	− 6.2 (− 9.9, − 2.3)	− 8.6 (− 7.9, − 7.5)	− 6.2 (− 2.9, − .0)	− 2.4 (− 6.6, − .0)	0.16	− 3.1 (− 1.9, − 3.3)	− 8.9 (− 2.5, − 1.3)	− 1.0 (− 2.3, − .8)	− 5.3 (− 3.5, − .0)	0.01
Occupation	− 6.2 (− 1.1, − 1.1)	− 3.3 (− 8.3, − .8)	− 6.7 (− 4.5, − 7.9)	− .5 (− 3.6, − .0)	0.01	− 1.9 (− 2.9, − .1)	− 5.1 (− 6.0, − 1.8)	− 9.5 (− 4.4, − .1)	− 1.4 (− 2.1, − 5.6)	0.37

Adjusted Model: Age, sex, baseline year, education, wealth index, country income level, chronic disease at baseline, BMI, and nested random intercept for each community by center. Change model includes baseline population density.

^†^*p* value for interaction between country income level and each urbanization metric.

^#^β coefficients represent percent change in MET minutes per week of total or domain specific physical activity.

### Associations between impervious area and physical activity

Table 4 summarizes models examining baseline impervious surface area and change in impervious surface area and total and domain specific PA. In fully adjusted models, an IQR increase in baseline impervious surface area was associated with a − 29.2% (95% CI − 37.5, − 19.7) lower population median of total PA, while an IQR increase in impervious surface area change (log transformed) was not associated with total PA. Impervious surface area change was associated with − 11.6% (95% CI − 21.7, − 0.1) lower recreational PA, but was not associated with any other PA outcome. The largest negative associations for baseline impervious surface area and PA were observed in LICs and for household PA, while positive associations were observed for transport and recreational PA in HICs and MICs. Statistically significant differences in the association of baseline impervious surface area and transport PA was observed across country-income status.

**Table 4:** Associations between impervious surface area at baseline, and 5-year impervious surface change prior to baseline, and individual-level physical activity.

Impervious area at baseline (β per IQR = 73%)						5-year change in impervious area (log) (β per IQR = 2% increase)				
	All	HIC	MIC	LIC	*p* value^†^	All	HIC	MIC	LIC	*p* value^†^
Total PA	− 9.2 (− 7.5, − 9.7)	− 0.6 (− 3.2, − .4)	− 1.8 (− 3.1, − .7)	− 4.4 (− 4.9, − 9.4)	0.01	2.0 (− .3, 12.2)	− .9 (− 3.0, 8.2)	1.5 (− 1.1, 16.1)	3.9 (− 4.6, 43.5)	0.98
Transport	67.2 (42.1, 96.8)	154.5 (88.9, 242.9)	102.7 (67.7, 144.9)	− 8.4 (− 8.6, − 3.4)	< 0.001	− .9 (− 3.5, 11.1)	− .5 (− 3.6, 9.5)	1.7 (− 4.4, 20.8)	− 0.4 (− 5.9, 25.1)	0.85
Recreation	54.9 (32.9, 80.8)	15.2 (− 1.6, 50.1)	81.3 (49.2, 120.3)	− 2.0 (− 2.6, 5.9)	0.30	− 1.6 (− 1.7, − .1)	2.6 (− 3.5, 21.9)	− 7.5 (− 0.5, − .0)	− .1 (− 7.8, 22.2)	0.14
Household	− 5.5 (− 2.3, − 7.7)	− 3.9 (− 5.4, − 9.5)	− 3.5 (− 2.2, − 3.3)	− 9.6 (− 1.8, − 2.4)	0.24	8.30 (− .11, 21.06)	6.7 (− .8, 23.5)	− .2 (− .8, − .5)	1.6 (− 7.0, 41.6)	0.55
Occupation	− 1.5 (− 9.3, − 1.6)	− 4.1 (− 0.5, − 2.2)	− 1.8 (− 1.5, − 9.6)	− 6.4 (− 7.0, − .1)	0.66	15.46 (1.17, 31.78)	25.6 (3.7, 52.2)	− .7 (− 9.9, 15.8)	17.1 (− 6.9, 65.2)	0.01

Adjusted Model: Age, sex, baseline year, education, wealth index, country income level, chronic disease at baseline, BMI, and nested random intercept for each community by center. Change model includes baseline impervious surface area.

^†^*p* value for interaction between country income level and each urbanization metric.

^#^β coefficients represent percent change in MET minutes per week of total or domain specific physical activity.

### Sub-group analysis of urbanization and physical activity

**Figure 2:**
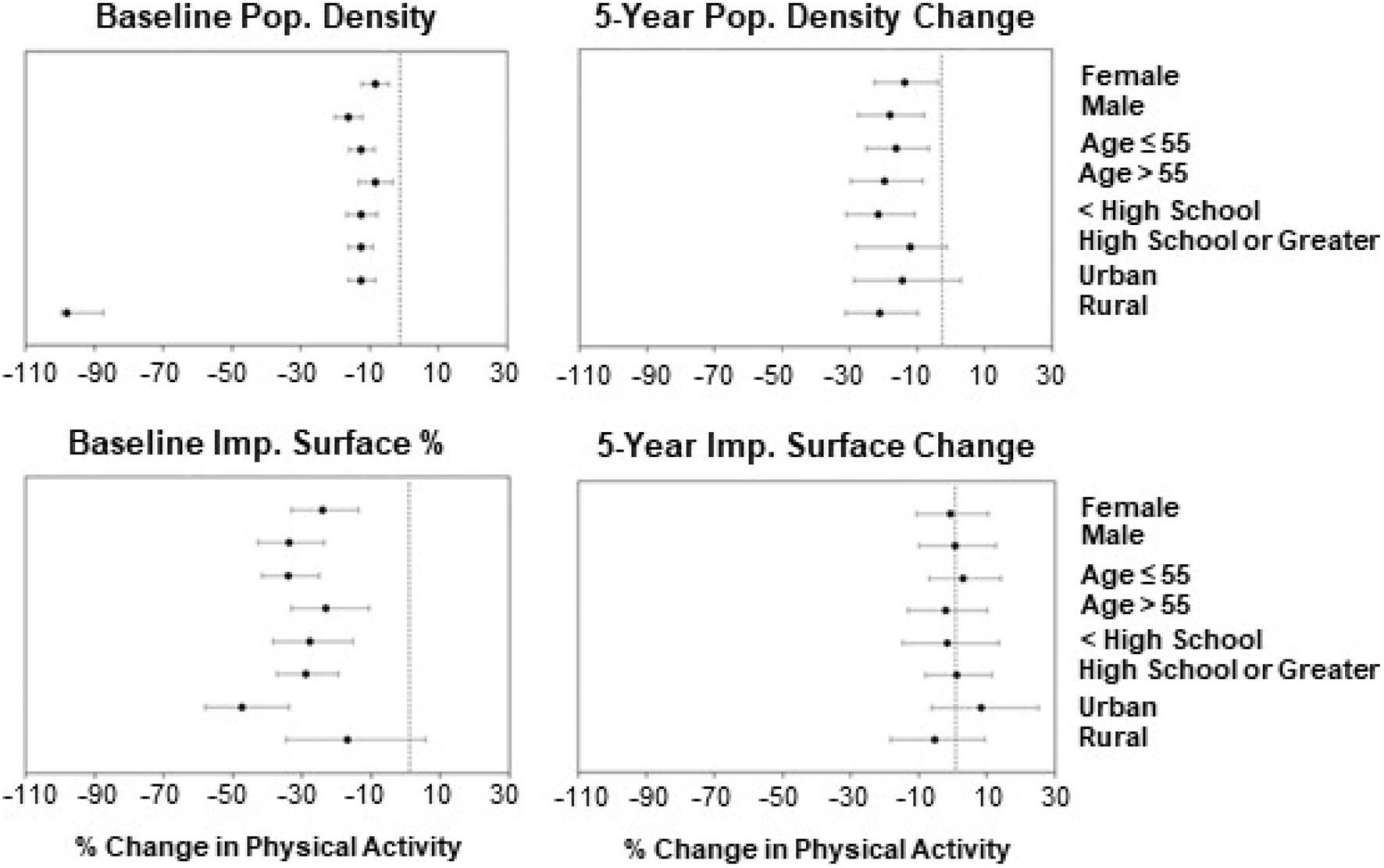
Adjusted associations between urbanization measures and total physical activity by sex, age, education and urban/rural categories.

We observed minimal differences in associations in our sub-group analyses by sex, age, and educational status, with some differences by urban/rural status. Figure 2 illustrated stratified models for total PA and our four urbanization measures. For example, an annual IQR increase in population density change over a 5 year period was associated with lower population median total PA among individuals living in rural areas (− 21.1%; 95% CI − 31.0, − 9.7) compared to urban areas (− 14.3%; 95% CI − 28.7,3.0); among males (− 18.0%; 95% CI − 27.6, − 7.8) compared to females (− 13.5%; 95% CI − 22.6, − 3.3); among individuals > 55 years (− 19.6%; 95% CI − 29.5, − 8.4) compared to individuals ≤ 55 years (− 16.2%; 95% CI − 24.9, − 6.5); and among individuals with < High School education (− 21.5%; 95% CI − 31.0, − 10.7) compared to individuals with high school or greater (− 12.1; 95% CI − 21.8, − 1.2). Similar trends are observed in these sub-group analyses when we stratified by HIC, LIC and MIC (Figs. S5–S19).

### Sensitivity analysis

Our model results were robust to sensitivity analyses. Adding country to our models to control for potential confounding in urbanization and PA levels between countries did not alter the results (Tables S3, S4). Models that included both population density and impervious surface area measures were consistent in terms of direction of association with minimal changes in the magnitude of associations compared to our main models (Tables S5, S6). When we used untransformed PA measures, as well as untransformed impervious surface area change, we observed similar associations and conclusions to our main models (Tables S7, S8).

## Discussion

This is the first study to examine associations between urbanization and different domains of PA for high-, middle-, and low-income countries using individual data on PA levels. Across all 698 communities from 22 countries, population density and impervious surface area were independently associated with lower total PA levels, with the largest inverse associations for household and occupational PA and small increased associations for transport and recreation PA. Change in population density levels for the 5 years prior to study baseline was also significantly associated with lower total PA, household PA, and occupational PA, but also increased associations for recreational and transport PA. Less robust associations were observed for change in community impervious surface area. Important differences in the associations between urbanization metrics and specific domains of PA were observed between country income levels, by urban and rural status, and sex.

Consistent with our results, other studies in HICs, MICs and LICs report that urbanization is associated with lower overall PA^17,36–39^, although there is substantial heterogeneity in findings as documented by recent systematic reviews^19,39^ . For example, a study in China, a MIC, reported community urbanization measures (increased population density, access to markets for household goods, economic wellbeing, transportation, communications, educational institutions, health facilities, sanitation and housing infrastructures) were associated with approximately 57% and 40% declines in total PA for men and women respectively over a 9 year period^17^. In Iran, a MIC, researchers found that increasing urbanization levels, measured using an index created from demographic, socioeconomic, and health-related variables, was associated with 7% and 2% higher odds of lower PA among males and females respectively^40^. A study in Kenya, a LIC, found that urbanization, measured using an urban–rural classification, was associations with lower overall PA levels among urban residents compared to rural counterparts^18^. While the measures of urbanization in these studies are very different, they suggest that overall, increasing urbanization is associated with decreased total PA, supporting the results of our study.

Importantly, different urbanization measures vary in their impact on specific domains of PA, and the magnitude of change also varies across high-, middle-, and low- income countries. These findings imply that policy makers globally need to address specific pathways and mechanisms by which urbanization is related to PA and that evidence from HICs cannot be applied directly to LICs. The larger reductions in occupational PA and household PA observed in our study in relation to the 5-year change in urbanization, especially among HICs and MICs compared to LICs, could be explained by the rapid changes in economic activity, socioeconomic conditions, occupational activities, and social, cultural and physical environments that occur with urbanization in LICs. Alternatively, recreation and transport PA was higher for some urbanization metrics, especially in HIC and MICs, and lower in LICs, suggesting potential mechanisms to increase total PA through urban planning that encourages walking and cycling. These differences could be attributed to changes in the built environment that promote transport PA, such as street connectivity, aesthetics, walking/cycling facilities and land use mix diversity in HICs and MICs^41–44^. The fact that urbanization in LICs was associated with decreases in transportation and recreation PA suggests more focused research is needed to understand how to optimize physical environments for PA during rapid urbanization.

We also explored differences by sub-group analysis based on socio-demographic characteristics. We found minimal to no differences between urbanization and PA across the different socio-demographic characteristics including age, and educational status. We found this trend to be consistent in the overall population as well as when stratified by country-income levels. In terms of urban–rural differences, we observed variation in the associations between urbanization measures and domain specific PA, especially for occupational and household PA in LICs. These findings align with other research that has shown that urbanization is associated with household and occupational PA differently in urban compared to rural areas^27,45,46^. In urban areas, urbanization may lead to an increase in sedentary jobs through technological advancements leading to less occupational and household PA. Our findings contribute to this literature and highlight important differences by rural–urban areas that should be considered when assessing how urbanization may be associated with the different domains of PA.

There is no universal definition or measurement method for urbanization, and a range of different metrics have been used to study associations between urbanization and PA or health^1,47,48^. Urbanization is an extremely complex phenomenon and different urbanization measures likely capture different aspect of the urban environment and associated change. In our study we observed moderate correlations between two measures (community population and impervious area) with PA, and these measures likely capture different components of urbanization (with impervious area capturing more development-related components of urbanization). Urbanization can also be context-specific; thus, an urbanization indicator may not necessarily capture the same construct in different communities (i.e. HIC, MIC or LIC country settings)^49^. Nevertheless, our two urbanization measures are objective measures that can be applied to any community, and are common urban metrics that capture large-scale upstream urban characteristics important to PA. Our results will help policy makers and urban planners understand how urbanization is associated with PA and ultimately how urbanization can be optimized to increase population PA levels. For example, our findings of different patterns of associations between urbanization and transport and recreational PA between LICs and MICs/HICs suggests that more attention is needed to optimize built environments for PA early in the urbanization process. Further, our findings better inform urban public health policy makers about the major health problems that may arise with urbanization in their regions without the required social supports and infrastructure changes to support overall population PA. This is especially important in rapidly urbanizing communities in developing countries.

While our study has several strengths, there are some potential limitations to highlight. First, this is a cross-sectional study, thus making it difficult to assess the causality of related factors. However, we used a 5-year change measure prior to enrollment to capture urbanization changes in each community. In addition, the PURE population is diverse and captures different populations and community settings, adding to the generalizability of our findings. Second, this study relies on self-reported measures of PA, which may be influenced by social desirability and recall biases. However, the use of self-reported PA estimates in this study has shown good validity and reliability against accelerometer data and other self-reported measures^50^. In addition, the inclusion of multiple domains of PA (transport, leisure, occupation and household) is unique and important for determining how urbanization is associated with different domains of PA, especially in developing countries where there is a paucity of studies. Third, we measured urbanization only using population density and impervious surface area. Other important dimensions of urbanization, such as of infrastructure, economic and demographic characteristics, were not directly measured. Nevertheless, these two objectively derived measures capture different aspects of urbanization that are important to PA, as well as for policy and planning. Finally, while we had detailed information on individual, household and community variables, there are unmeasured factors that are likely important to our analyses. For example, sociocultural norms may discourage outdoor PA for women aside from within the home (household PA) or built environment design may influence the amount of transport of recreation PA available^51,52^. While these factors are important and will be examined in future research, here we examined the potential impact of these unmeasured factors on our analysis using community and center random effects, which did not change our over-all results.

## Conclusions

Urbanization was associated with lower total PA in this diverse global study. Lower household and occupational PA were most strongly associated with urbanization, especially in LICs and MICs. In HICs, urbanization was associated with higher recreation PA. Important differences in the associations between urbanization metrics and PA were observed between country income levels, as well as by urban and rural status and sex, which suggest that research from HICs cannot be directly applied to LMICs. These findings provide new information on the complex associations between urbanization and PA.

## Methods

### Study design and participants

The overall PURE study consists of approximately 183,503 adults aged 35–70 years residing in 800 urban and rural communities in 27 countries encompassing great sociocultural diversity^53^. Recruitment commenced in 2003 and follow-up is on-going. Participants were sampled from communities, representing neighborhoods in urban areas and small villages in rural areas. Within each defined community, households were approached, and individuals aged 35–70 years who intended to live at their current address for at least another 4 years were invited to participate in the study. Baseline data collection occurred at the community, household and individual levels. Variables such as sociodemographic factors, medical history, lifestyle behaviors, and risk factors including baseline chronic diseases were recorded in the PURE study using standardized measures and procedures. Detailed methodology and design of the PURE study have been described elsewhere^54,55^. All participants in the PURE study provided written informed consent. The study was coordinated by the Population Health Research Institute (Hamilton Health Sciences, Hamilton, ON, Canada) and approved by the Hamilton Health Sciences Research Ethics Board and local ethics committees at each center. The study protocol conformed to the ethical guidelines as stated in the Declaration of Helsinki.

Here we restrict to the 138,206 adults with complete PA outcomes assessed at baseline using the long-form International PA Questionnaire (IPAQ). Our study population represents individuals from 698 communities located in 22 countries, including 5 low income countries (LICs) (Bangladesh, India, Pakistan, Tanzania, and Zimbabwe); 13 middle income countries (MICs)(Argentina, Brazil, Chile, China, Colombia, Iran, Malaysia, Palestine, Philippines, Poland, Russia, South Africa, and Turkey); and 4 high income countries (HICs) (Canada, Saudi Arabia, Sweden, and the United Arab Emirates)^53^. The countries are grouped into economic levels based on World Bank classifications at study baseline^56^. The choice and number of countries selected in PURE reflects a balance involving many countries at different economic levels with substantial heterogeneity in social and economic circumstances.

### Physical activity levels

PA outcomes were assessed using the long-form IPAQ. Our outcome included the four domains of PA (occupational, transportation, housework, recreational) and total PA reported in metabolic equivalents (MET) × minutes per week (MET-minutes per week). Participants were asked to report frequency and duration of specific activities including vigorous and moderate intensity physical activities, and walking in terms of the frequency and duration in the last 7 days. For each individual, the recorded activities were converted to MET-minutes per week using equations adapted from the IPAQ data processing guidelines^57^. Specific rules for screening outliers are documented in the IPAQ data screening method. A summary of the final equations used in deriving PA is presented in Table S2.

### Urbanization measures

Measuring the degree of urbanization is challenging due to varying definitions, especially across different countries and settings^58^. Urbanization has been measured using a variety of methods, such as population size and density, land, economy, society and ecologic variables^1,47^, or the presence of impervious surfaces or built up areas^59^. Impervious area refers to impervious materials such as concrete, metal, glass, tarmac, and plastic materials that cover the soil and serve as an important factor for the ecological impact of the built environment^60^. For the purposes of this study, population density and impervious surface area were used as our primary measures of urbanization, as these two measures capture different urbanization dimensions that can be objectively measured with satellite data using similar metrics across diverse communities and countries.

#### Population density

We used the fourth version of the Gridded Population of the World. (GPWv.4)^63^ to measure population density for all PURE community locations. The GPWv4 models the distribution of human population (counts and densities) using sub-national population estimates from around the world, which are derived using periodic census data for each country and is gridded with an output resolution of 30 arc-seconds (approximately 1 km at the equator) and is available for the years 2000, 2005, 2010, and 2015. We calculated population density within 1 and 5 km buffer regions for each PURE community centroid. We examined the 1 km buffer region as a measure of local community change and the 5 km buffer as a measure of larger-scale urbanization in an area.

We examined two primary urbanization measures using population density. The first is baseline population density for each PURE community at the time the IPAQ questionnaires were completed. We assigned the closest GPWv4 year to each year of enrollment in the PURE study (2003–2015). The second is the 5-year annual population density rate change (Eq. 1 below) prior to study baseline in each community. We use this equation to calculate the average annual change in population density per year to be consistent with other studies of urbanization^62–64^.

1 $$ {\text{5-year}}\;{\text{Population}}\;{\text{Density}}\;{\text{Change}}\;{\text{Rate }} = \frac{{{\text{PD}}_{{\text{e}}} - {\text{PD}}_{{\text{p}}} }}{{{\text{PD}}_{{\text{p}}} }} - \frac{1}{5}*100 $$

where PD_p_ = Population density 5 year prior to PURE enrollment datePD_e =_ Population density at PURE enrollment date.

#### Impervious area

We examined impervious area as another measure of urbanization. Impervious area is related to population density but captures a distinct component of urbanization related to land use/cover change^65,66^. We derived impervious area metrics for each PURE community using the global artificial impervious area (GAIA) dataset^67^. Briefly, GAIA is a global dataset that captures the growth of artificial impervious areas annually from 1985 to 2018. GAIA is made up of mapped annual artificial impervious area at a 30 m resolution from the full archive of Landsat images. The modelling approach to capturing impervious area demonstrated a mean overall accuracy over multiple years greater than 90%^67^.

Similar to population density, we calculated the percent of impervious area within a 1 km and 5 km buffer regions for each PURE community centroid. We assigned the corresponding impervious surface area to each year of enrollment in the PURE study (2003–2015). We examined two primary urbanization measures using impervious area, similar to the approach used for population density. The first is baseline percent impervious area for each PURE community. The second is the 5-year impervious area rate change (Eq. 2 below). Because the 5-year impervious area rate change was highly skewed, we use the log transformed measure in all analyses.

2 $$ {\text{5-year}}\;{\text{Impervious}}\;{\text{Surface}}\;{\text{Area}}\;{\text{Change}}\;{\text{Rate }} = \frac{{{\text{IS}}_{{\text{e}}} - {\text{IS}}_{{\text{p}}} }}{{{\text{IS}}_{{\text{p}}} }} - \frac{1}{5}*100 $$

where IS_p_ = Impervious Surface Area 5 year prior to PURE enrollment date. IS_e_ = Impervious Surface Area at PURE enrollment date.

### Statistical analysis

We first report descriptive statistics (e.g., mean, frequency, standard deviation) of all PA outcomes, urbanization measures and covariates. We excluded individuals with missing key continuous covariates and for missing categorical variables included a missing data category. Next, we examined correlations between all urbanization measures using Pearson’s correlations. We modelled cross-sectional associations between each urbanization measures and each PA outcome (total, leisure, transport, occupational, household) using generalized mixed effects models with a nested random intercept for communities by center to account for the clustered sampling design of the PURE study. We present adjusted models that include age, sex, baseline year, household wealth index, education, BMI, chronic disease status at baseline, country income level and a nested random intercept for communities by center. Covariates were determined a priori that may be related to urbanization and PA but not directly on the causal pathway. For models examining change in population density and impervious surface area we also include the baseline population density or impervious area in the models to control for differences in baseline urbanization levels. We ran overall models and models stratified by country income levels (HIC, UMIC, LMIC, LIC). Finally, we examined models stratified by key individual variables, including age (< 55, > 55), sex (male, female), educational status (< high school, > high school) and urban/rural status.

To account for residual non-normality distributions of the models and to describe the full distribution of PA data, we used log transformed PA outcome variables in all analyses using y = ln(y + 1). To ease interpretation of our model results, we exponentiated the coefficient, subtracted one from this number, and multiplied by 100. This gives the percent increase (or decrease) in the response variable. We present associations for interquartile range (IQR) increases in our urbanization metrics and the corresponding percent change in the population median of our PA measures. Thus, in the present study, percent change was approximated by setting both random effects equal to zero and using ln(y) as an approximation to ln(y + 1). We examined quartiles for total PA and the domains of PA, which did not demonstrate statistically significant departures from a linear relationship and therefore present linear results for all models.

We conducted several sensitivity analyses to evaluate the robustness of our main model results. First, we examined joint models that included both population density and impervious surface area measures to determine if these urbanization metrics have independent associations with PA. Second, we examined adding country to our models to control for potential confounding in urbanization and PA levels between countries. Third, we examined model results using un-transformed PA measures. All statistical analyses were performed using SAS® software 9.4^68^. We present results for fully adjusted models in our tables.

### Supplementary Information

Supplementary Information.

## Data Availability

The datasets generated and/or analyzed during the current study are not publicly available due to the confidential nature of the PURE health data but are available from the corresponding author on reasonable request.
