# Supplementary material for: Urbanization and physical activity in the global Prospective Urban and Rural Epidemiology study
Source: Sci Rep. 2023 Jan 6;13:290. doi: 10.1038/s41598-022-26406-5 (PMC9822998; doi:10.1038/s41598-022-26406-5)
Supplement: Supplementary file 1 — Supplementary Information. [file 41598_2022_26406_MOESM1_ESM.docx]

**Supplementary Files**


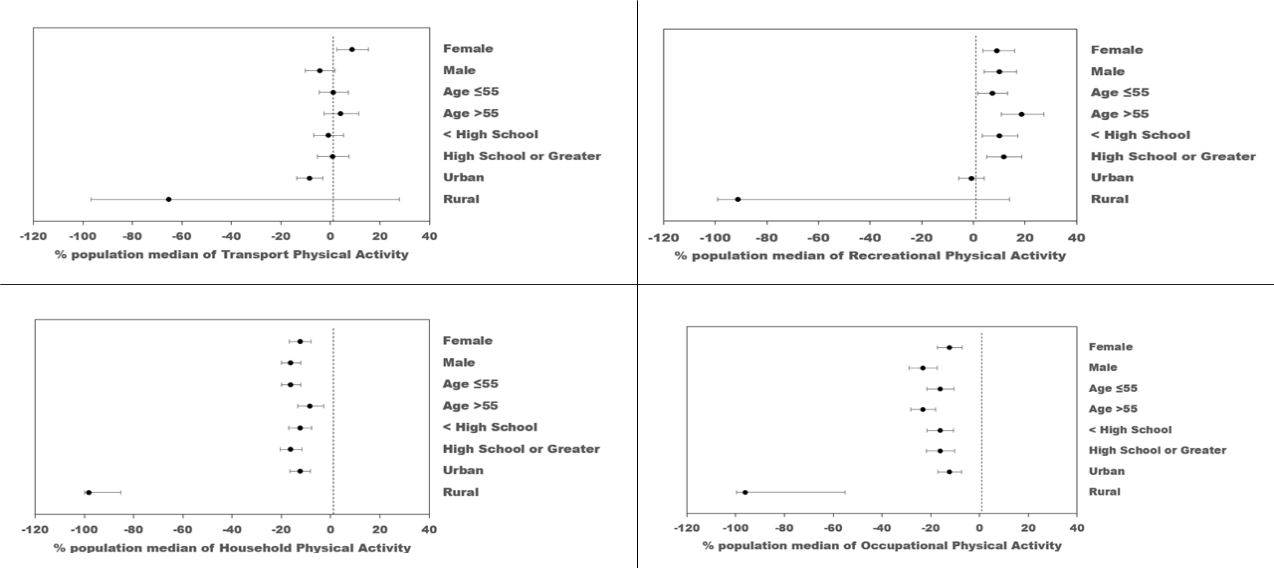


Figure S1. Adjusted associations between baseline population density and domain specific physical activity by sex, age, education and urban/rural categories for 138,206 adults participating in 698 communities across 22 countries in PURE


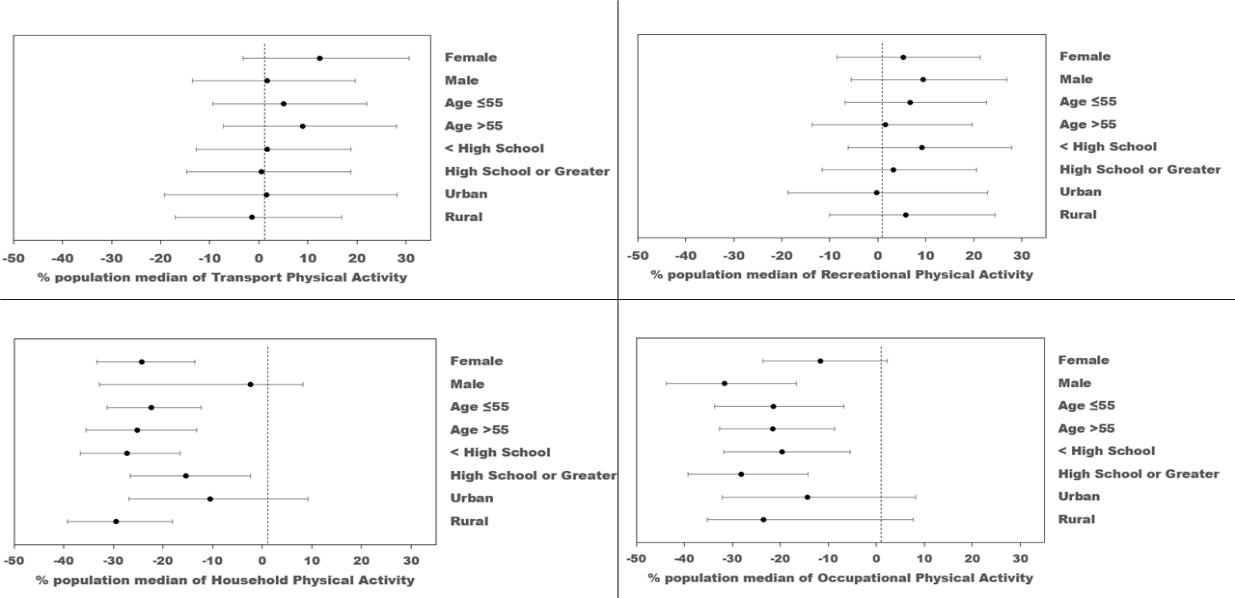


Figure S2. Adjusted associations 5-year change population density and domain specific physical activity by sex, age, education and urban/rural categories for 138,206 adults participating in 698 communities across 22 countries in PURE


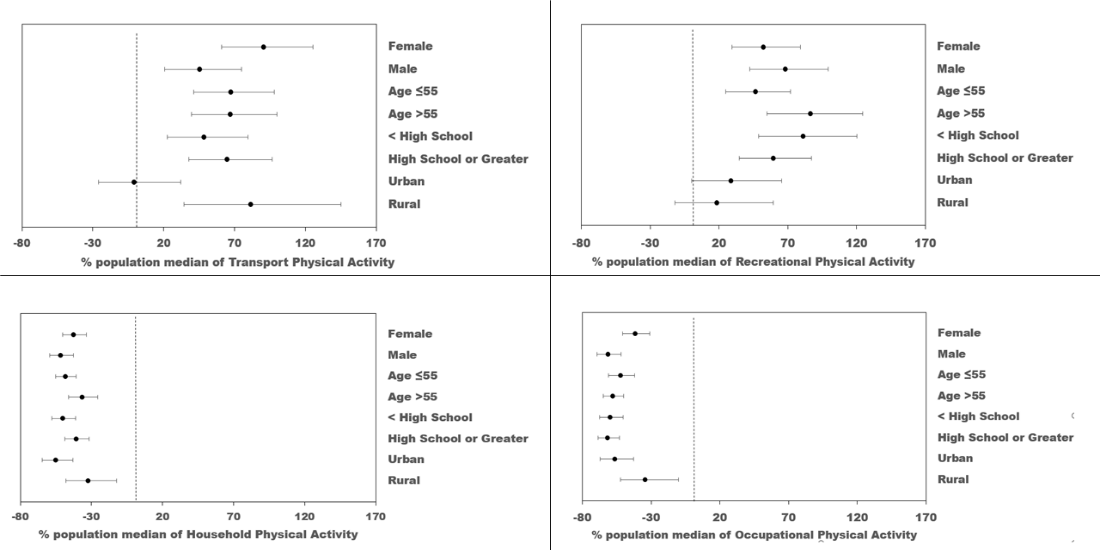


Figure S3. Adjusted associations between baseline impervious area and domain specific physical activity by sex, age, education and urban/rural categories for 138,206 adults participating in 698 communities across 22 countries in PURE


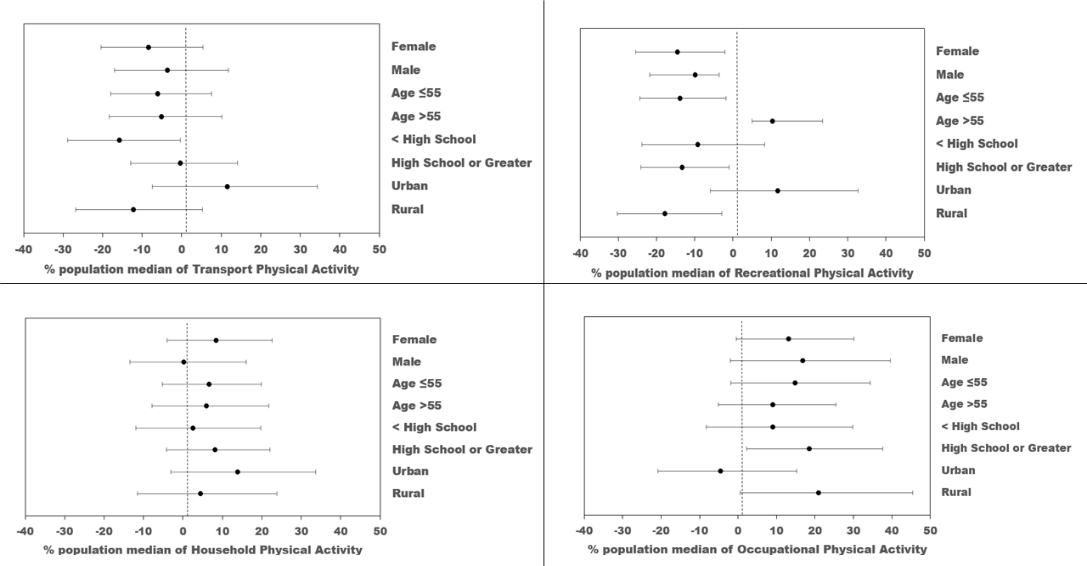


Figure S4. Adjusted associations between 5-year change impervious area and domain specific physical activity by sex, age, education and urban/rural categories for 138,206 adults participating in 698 communities across 22 countries in PURE


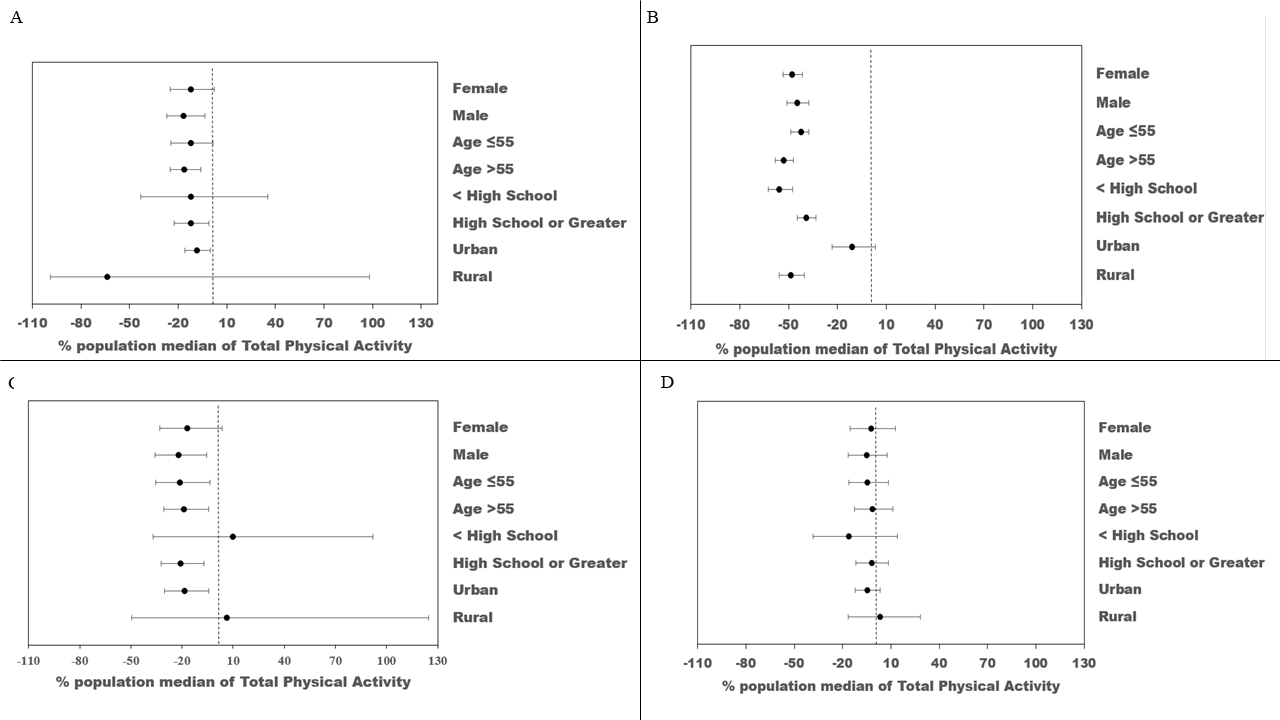


A = Baseline population density

B = 5-year population density Change rate (%)

C= Baseline impervious area (%)

D =5-year impervious area change rate (log-transformed)

Figure S5. Adjusted associations between baseline and 5-year change urbanization measures and total physical activity by sex, age, education and urban/rural categories for adults participating in High Income Countries in PURE


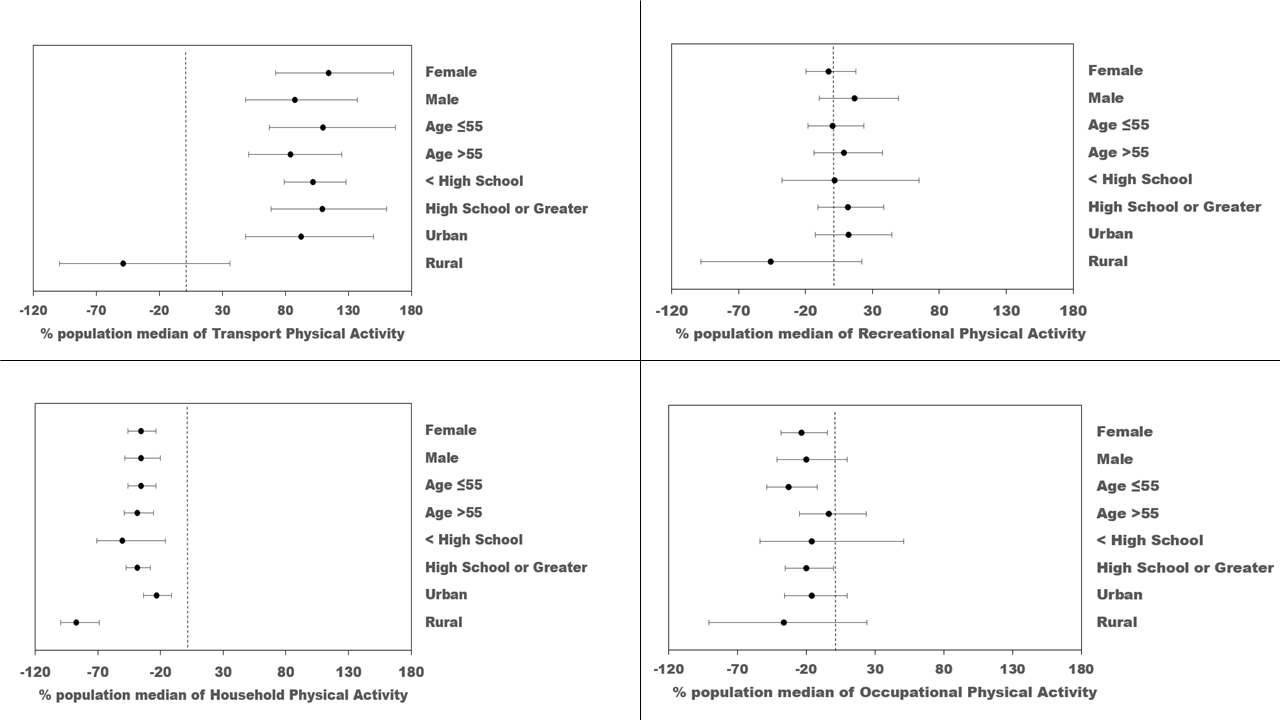


Figure S6. Adjusted associations between baseline population density and domain specific physical activity by sex, age, education and urban/rural categories for adults participating in High Income Countries in PURE


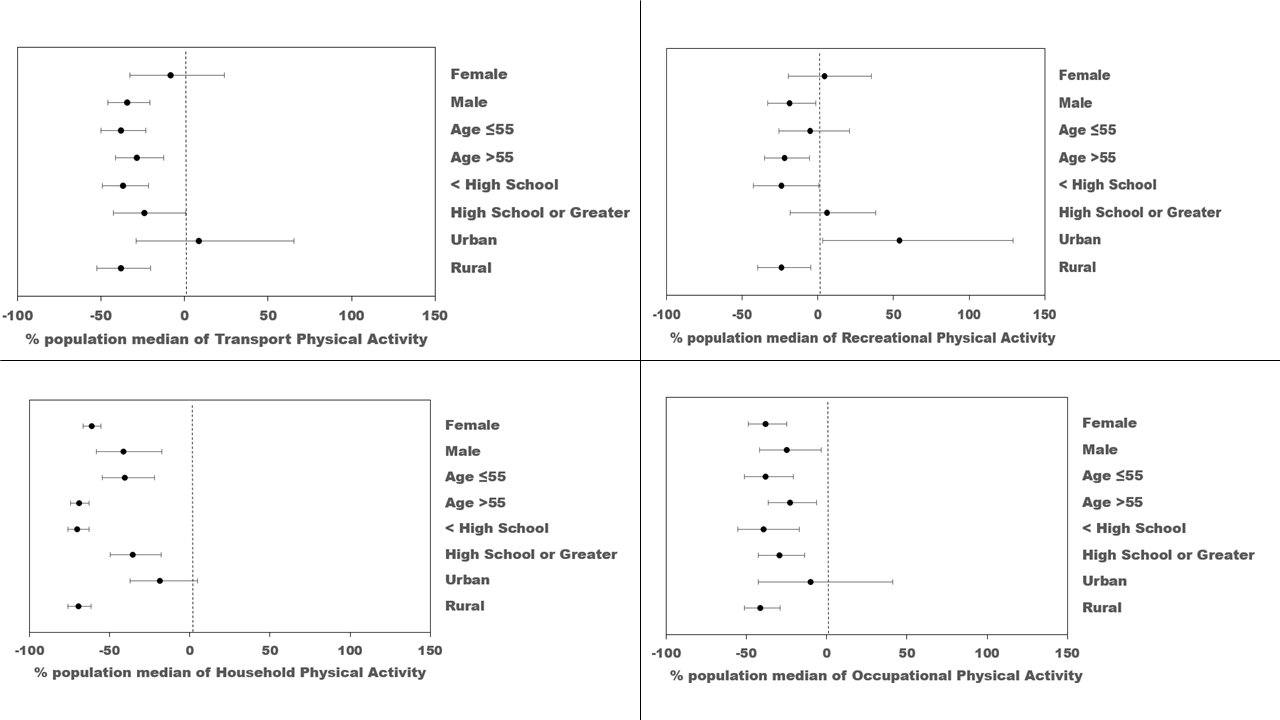


Figure S7. Adjusted associations between 5-year change population density and domain specific physical activity by sex, age, education and urban/rural categories for adults participating in High Income Countries in PURE


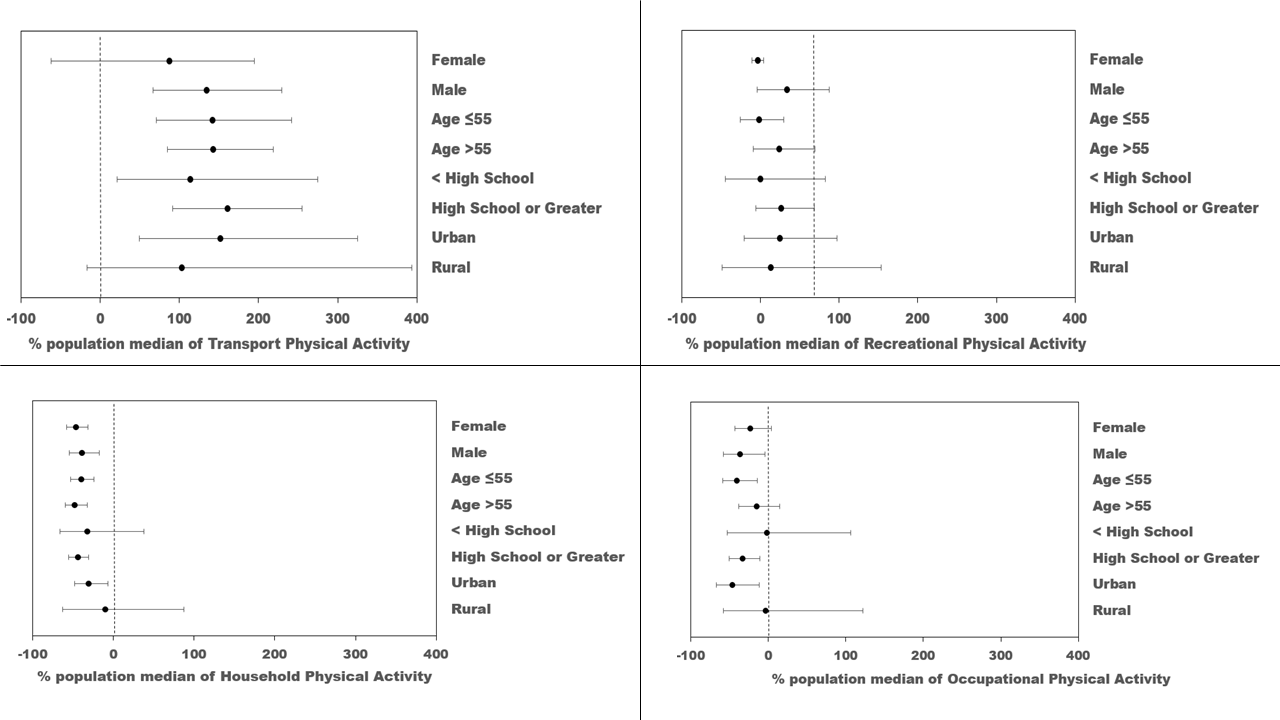


Figure S8. Adjusted associations between baseline impervious area and domain specific physical activity by sex, age, education and urban/rural categories for adults participating in High Income Countries in PURE


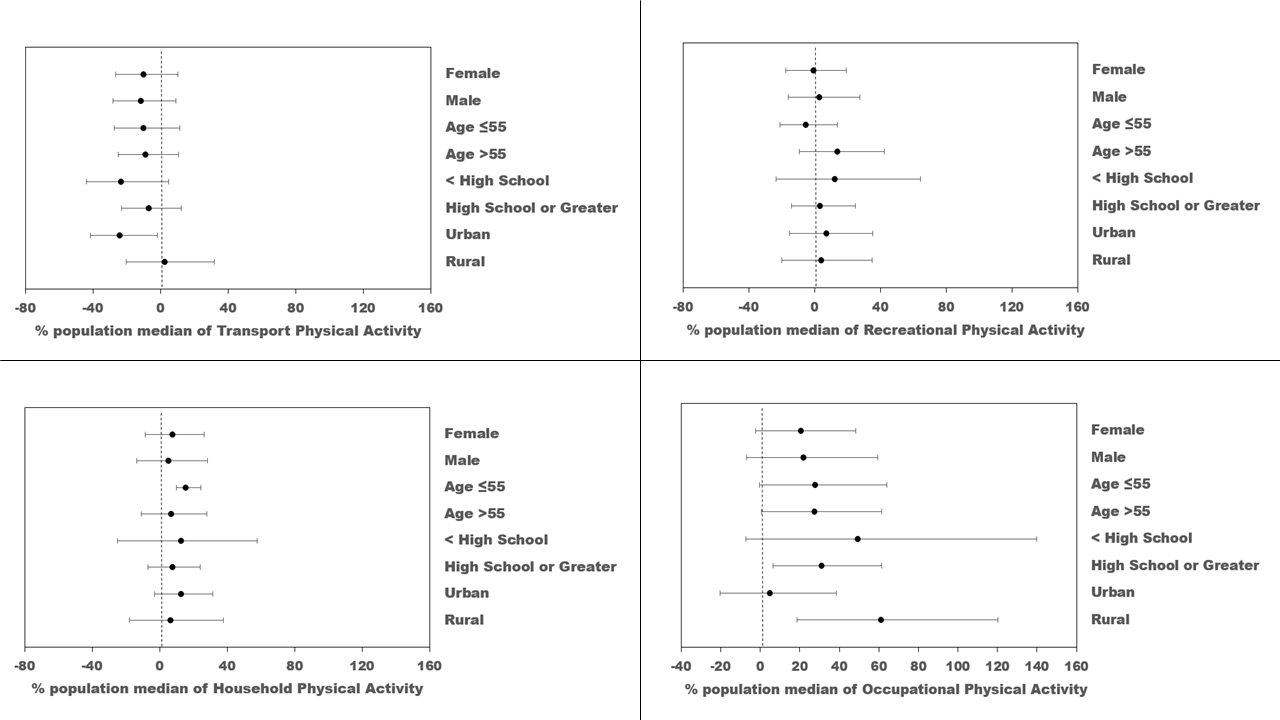


Figure S9. Adjusted associations between 5-year change impervious area and domain specific physical activity by sex, age, education and urban/rural categories for adults participating in High Income Countries in PURE


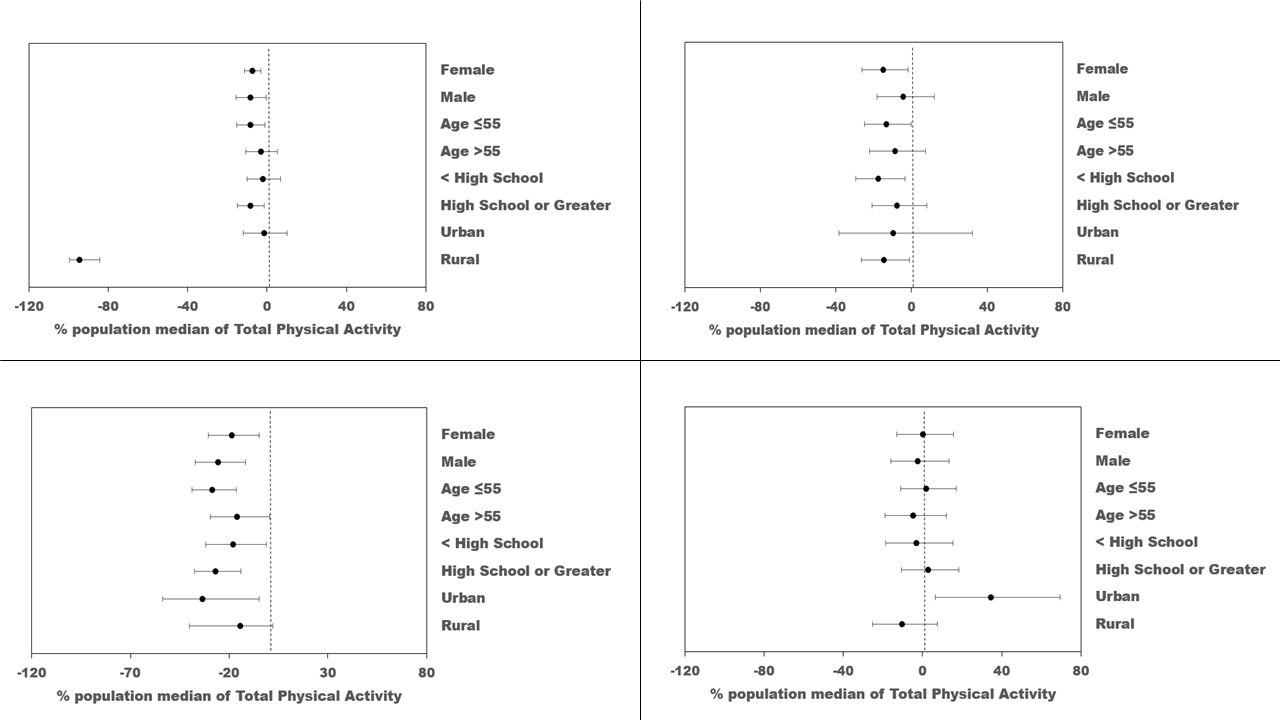


A = Baseline population density

B = 5-year population density Change rate (%)

C= Baseline impervious area (%)

D =5-year impervious area change rate (log-transformed)

Figure S10. Adjusted associations between baseline and 5-year change urbanization measures and total specific physical activity by sex, age, education and urban/rural categories for adults participating in Middle Income Countries in PURE


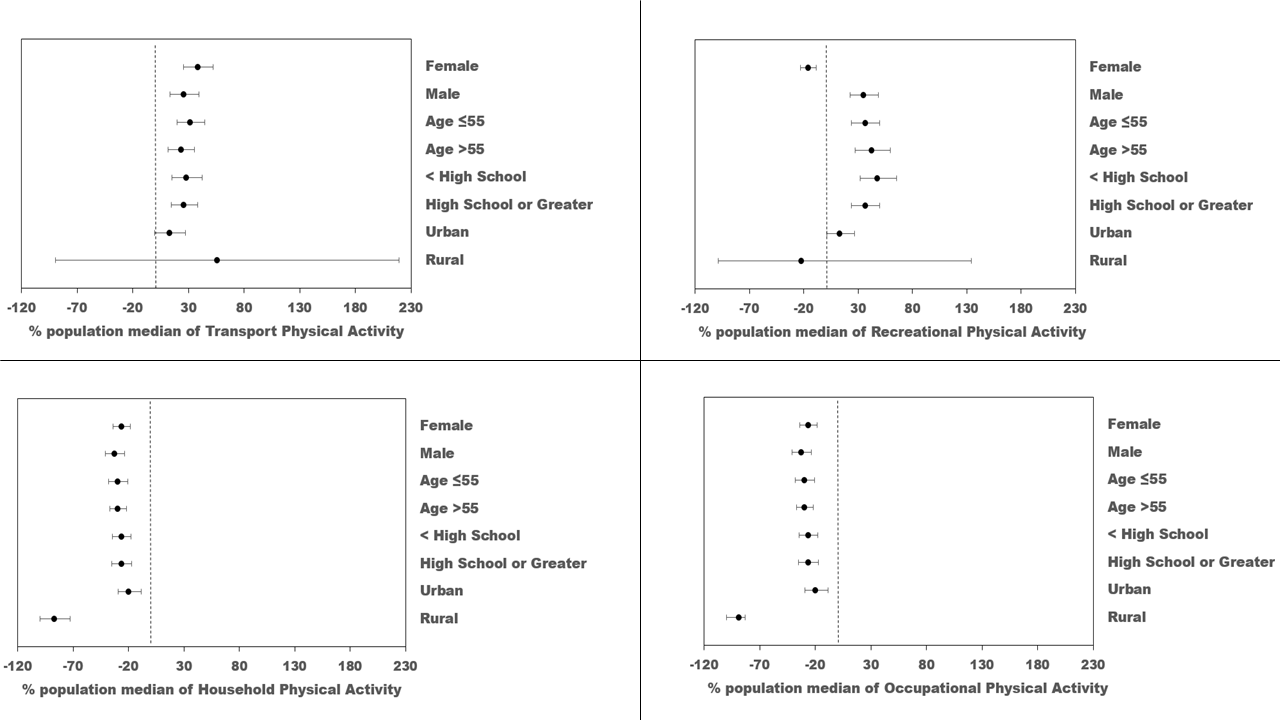


Figure S11. Adjusted associations between baseline population density and domain specific physical activity by sex, age, education and urban/rural categories for adults participating in Middle Income Countries in PURE


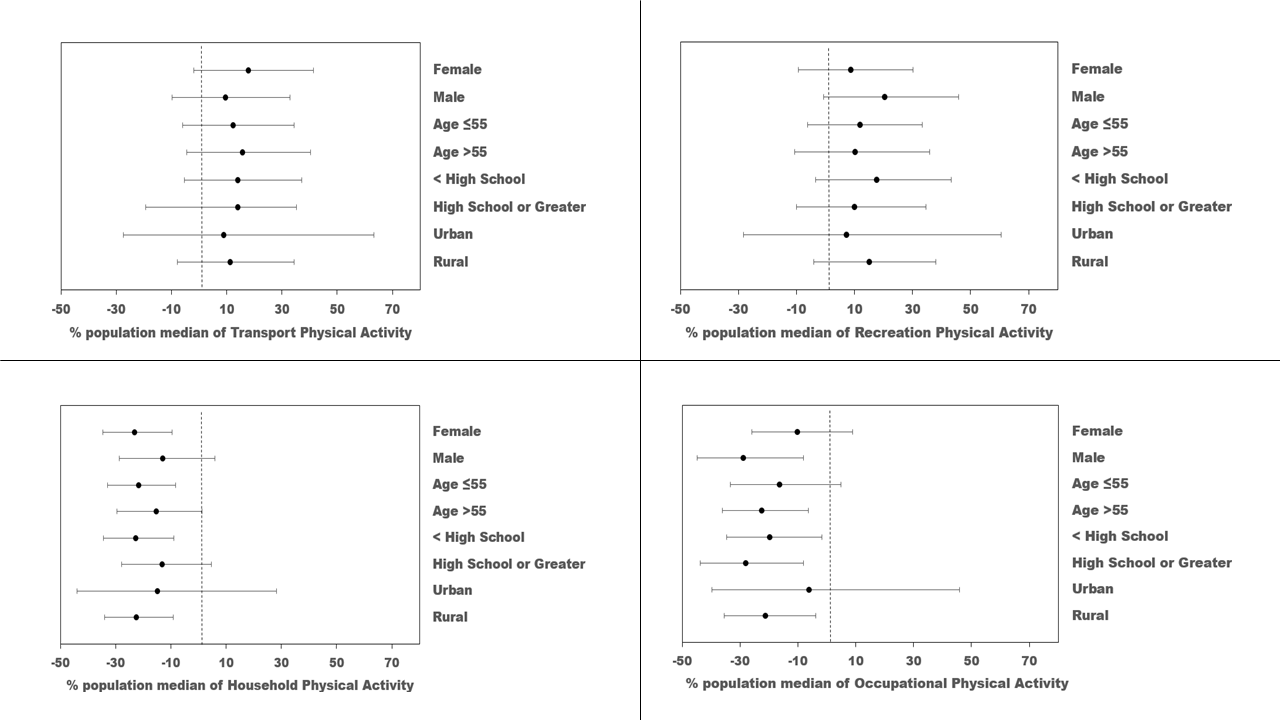


Figure S12. Adjusted associations between 5-year change population density and domain specific physical activity by sex, age, education and urban/rural categories for adults participating in Middle Income Countries in PURE


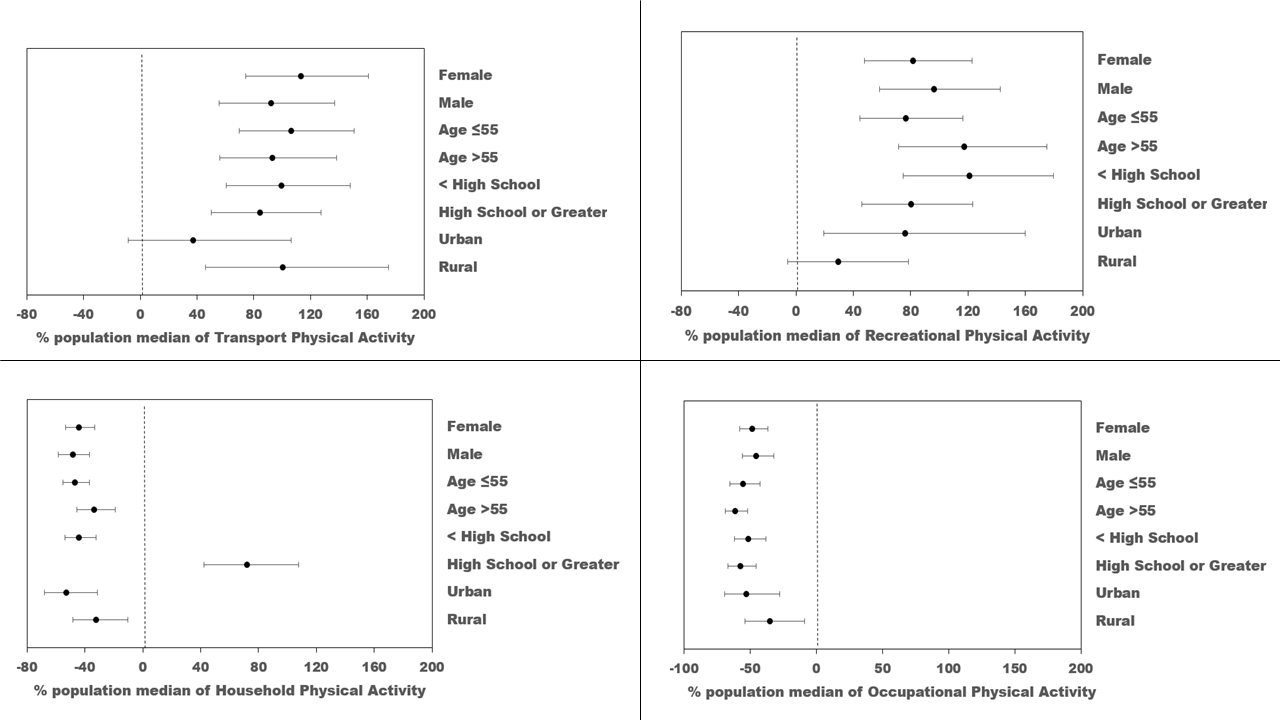


Figure S13. Adjusted associations between baseline impervious area and domain specific physical activity by sex, age, education and urban/rural categories for adults participating in Middle Income Countries in PURE


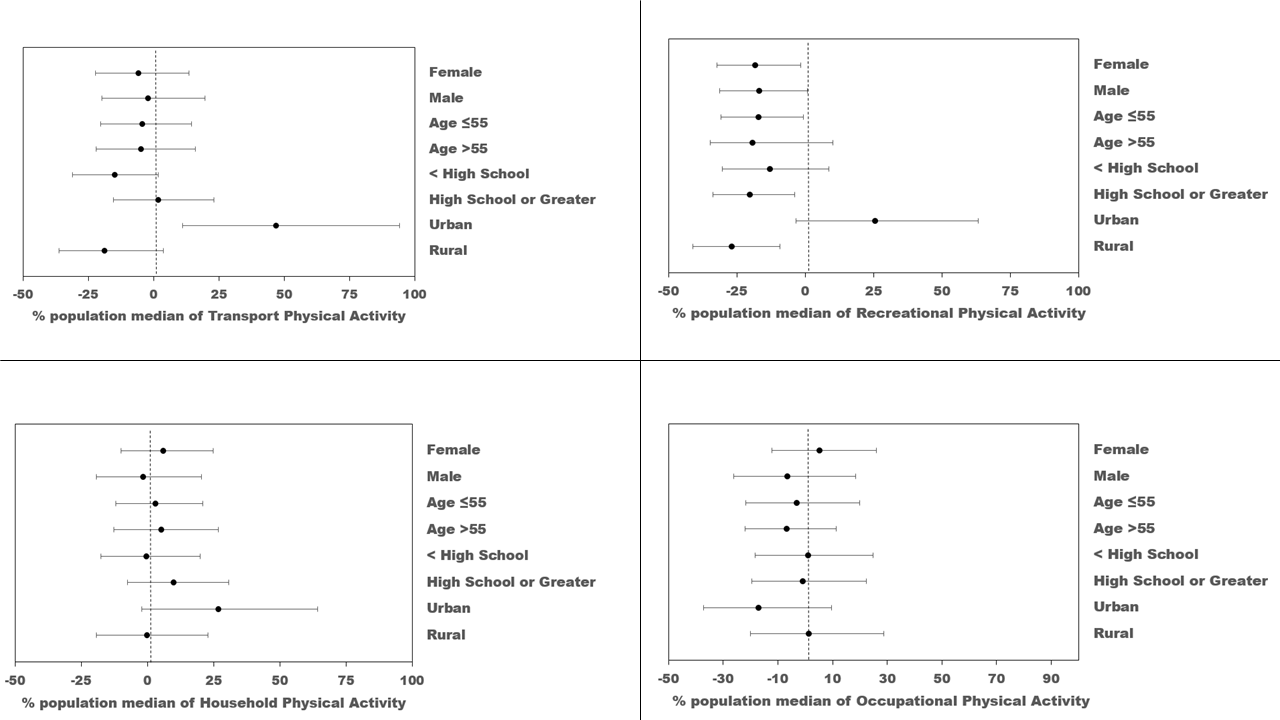


Figure S14. Adjusted associations between 5-year change impervious area and domain specific physical activity by sex, age, education and urban/rural categories for adults participating in Middle Income Countries in PURE


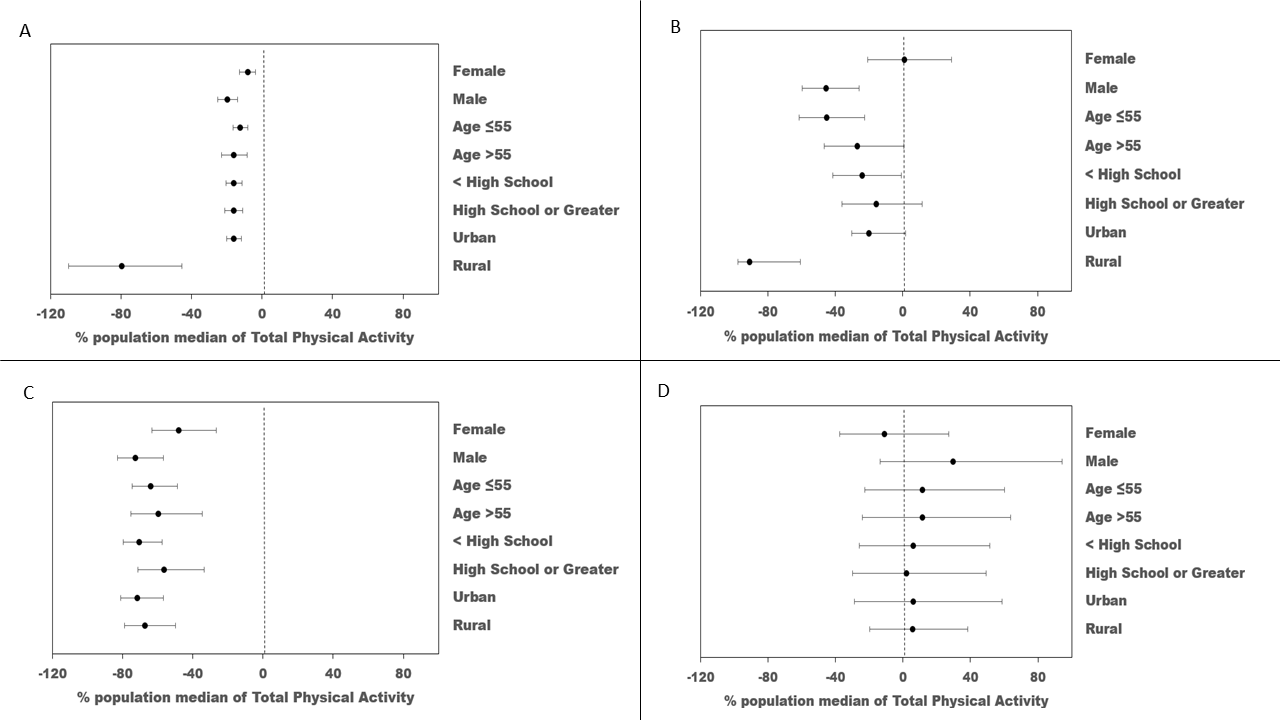


A = Baseline population density

B = 5-year population density Change rate (%)

C= Baseline impervious area (%)

D =5-year impervious area change rate (log-transformed)

Figure S15. Adjusted associations between baseline and 5-year change urbanization measures and total specific physical activity by sex, age, education and urban/rural categories for adults participating in Low Income Countries in PURE


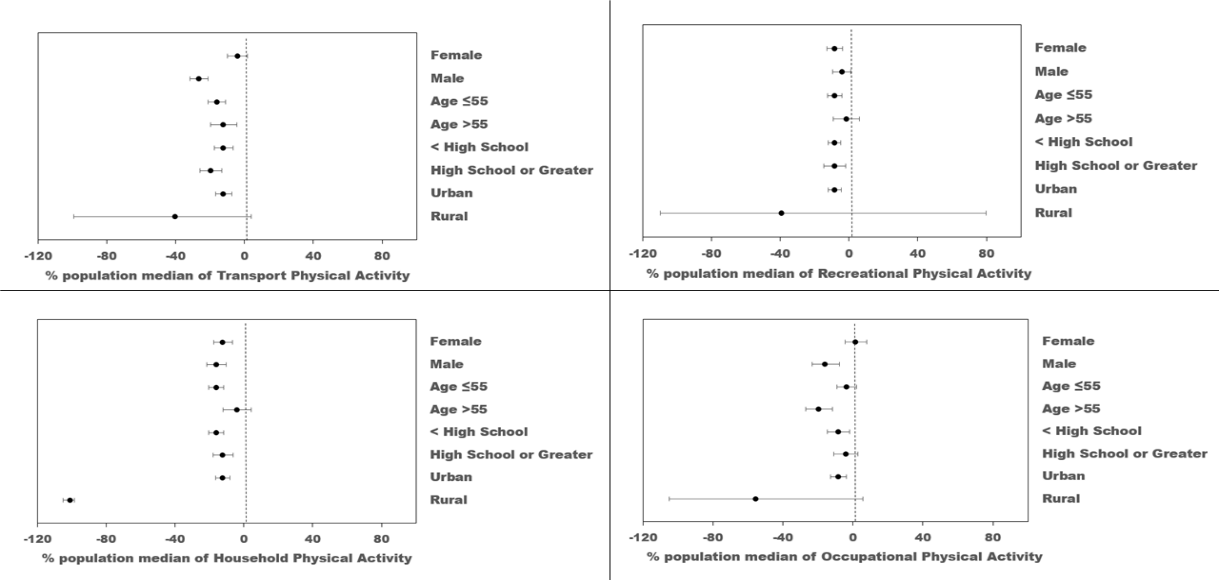


Figure S16. Adjusted associations between baseline population density and domain specific physical activity by sex, age, education and urban/rural categories for adults participating in Low Income Countries in PURE


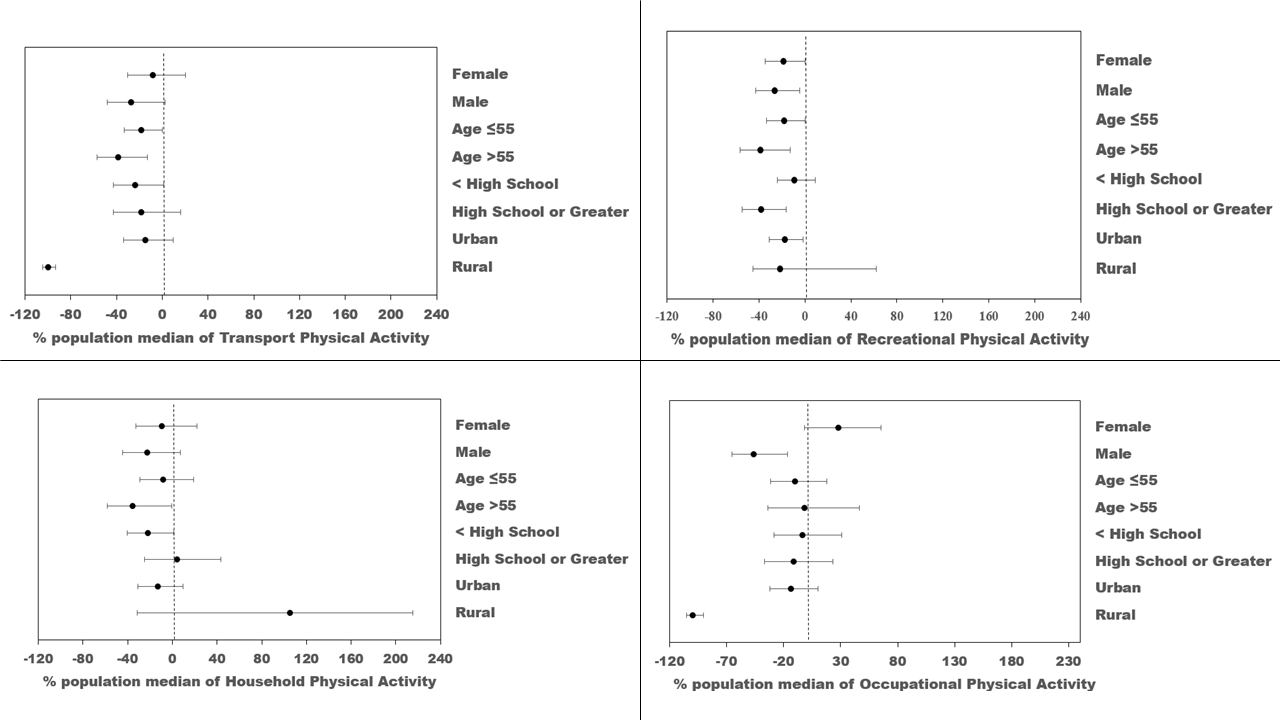


Figure S17. Adjusted associations between 5-year change population density and domain specific physical activity by sex, age, education and urban/rural categories for adults participating in Low Income Countries in PURE


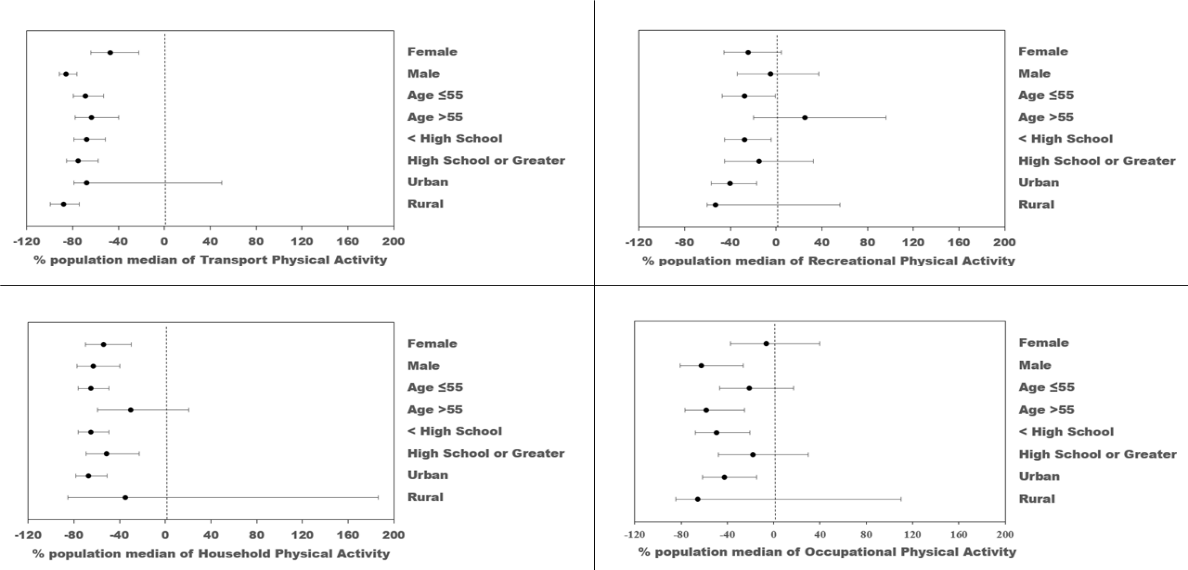


Figure S18. Adjusted associations between baseline impervious area and domain specific physical activity by sex, age, education and urban/rural categories for adults participating in Low Income Countries in PURE


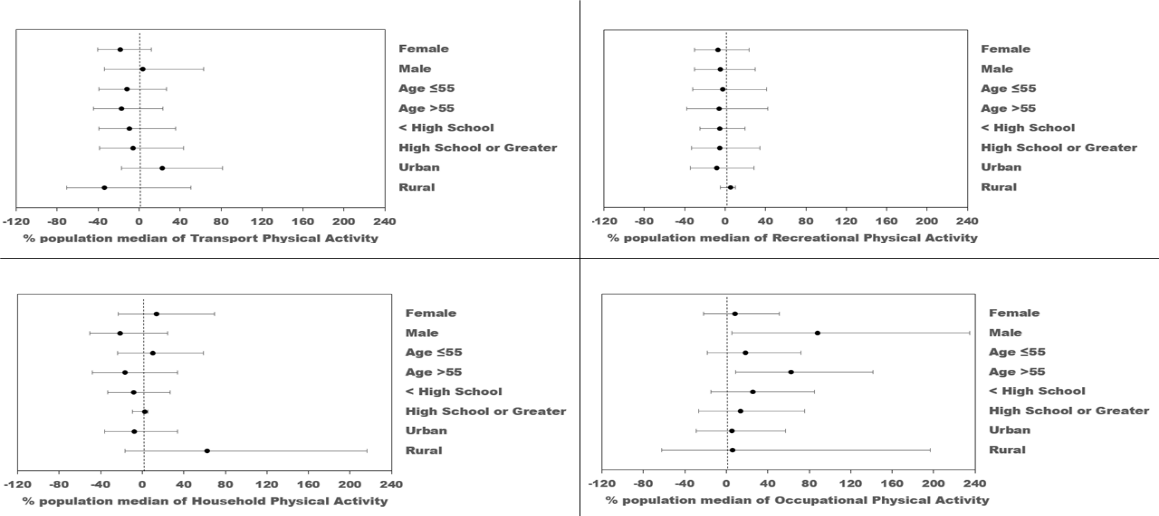


Figure S19. Adjusted associations between 5-year change impervious area and domain specific physical activity by sex, age, education and urban/rural categories for adults participating in Low Income Countries in PURE

Table S1. Correlation of urbanization measures across 698 communities across 22 countries in PURE.

|  | PD 1km | %IA 1km | ΔPD  1km  10yr | ΔPD  1km  5yr | ΔIA  1km  5yr | ΔIA  1km  10yr | PD 5km | %IA 5km | ΔIA  5km  5yr | ΔPD  5km  5yr | ΔPD  5km  10yr | ΔIA  5km  10yr |
| --- | --- | --- | --- | --- | --- | --- | --- | --- | --- | --- | --- | --- |
| PD  1km | 1.00 | 0.53 | -0.18 | -0.14 | -0.43 | -0.43 | 0.93 | 0.61 | -0.29 | -0.03 | -0.01 | -0.37 |
| %IA 1km | 0.53 | 1.00 | -0.08 | -0.01 | -0.55 | -0.56 | 0.56 | 0.89 | -0.39 | -0.01 | 0.01 | -0.43 |
| ΔPD1km10yr | -0.18 | -0.08 | 1.00 | 0.93 | 0.22 | 0.13 | -0.17 | -0.05 | 0.07 | -0.04 | 0.03 | 0.09 |
| ΔPD1km5yr | -0.14 | -0.01 | 0.93 | 1.00 | 0.26 | 0.15 | -0.08 | 0.04 | 0.13 | 0.02 | 0.04 | 0.13 |
| ΔIA 1km 5yr | -0.43 | -0.55 | 0.22 | 0.26 | 1.00 | 0.91 | -0.41 | -0.48 | 0.86 | 0.04 | 0.03 | 0.81 |
| ΔIA1km10yr | -0.43 | -0.56 | 0.13 | 0.15 | 0.91 | 1.00 | -0.42 | -0.51 | 0.77 | 0.03 | 0.03 | 0.86 |
| PD 5km | 0.93 | 0.56 | -0.17 | -0.08 | -0.41 | -0.42 | 1.00 | 0.66 | -0.30 | -0.03 | -0.03 | -0.38 |
| %IA 5km | 0.61 | 0.89 | -0.05 | 0.04 | -0.48 | -0.51 | 0.66 | 1.00 | -0.38 | -0.09 | -0.11 | -0.47 |
| ΔIA 5km 5yr | -0.29 | -0.39 | 0.07 | 0.13 | 0.86 | 0.77 | -0.30 | -0.38 | 1.00 | 0.01 | 0.00 | 0.88 |
| ΔPD 5km 5yr | -0.03 | -0.01 | -0.04 | 0.02 | 0.04 | 0.03 | -0.03 | -0.09 | 0.01 | 1.00 | 0.98 | 0.02 |
| ΔPD 5km10yr | -0.01 | 0.01 | 0.03 | 0.04 | 0.03 | 0.03 | -0.03 | -0.11 | 0.00 | 0.98 | 1.00 | 0.04 |
| ΔIA 5km 10yr | -0.37 | -0.43 | 0.09 | 0.13 | 0.81 | 0.86 | -0.38 | -0.47 | 0.88 | 0.02 | 0.04 | 1.00 |

PD 1km = baseline population density within a 1km buffer radius

PD 5km = baseline population density within a 5km buffer radius

%IA 5km =percent baseline impervious area within a 5km buffer radius

%IA 1km =percent baseline impervious area within a 1km buffer radius

ΔPD 1km_5yr = 5-year population density change rate within a 1 km buffer radius

ΔPD 5km_5yr = 5-year population density change rate within a 5 km buffer radius

ΔPD 1km_10yr = 10-year population density change rate within a 1 km buffer radius

ΔPD 5km_10yr = 10-year population density change rate within a 5 km buffer radius

ΔIA 1km_10yr =10-year impervious area change rate within a 1 km buffer radius(log-transformed)

ΔIA 5km_10yr =10-year impervious area change rate within a 5 km buffer radius(log-transformed)

ΔIA 1km_5yr =5-year impervious area change rate within a 1 km buffer radius(log-transformed)

ΔIA 5km_5yr =5-year impervious area change rate within a 5 km buffer radius(log-transformed)

Table S2. IPAQ MET Values and Formula for Computation of MET-minutes.

| Work PA | Walk at work (METs*min*days) + Moderate PA at work (METs*min*days) + Vigorous PA at work (METs*min*days) |
| --- | --- |
| Transportation PA | Walk for transportation (METs*min*days) + Cycle for transportation (METs*min*days). |
| Household PA | vigorous yard work (METs*min*days) + Moderate yard work (METs*min*days) + Moderate inside chores (METs*min*days) |
| Leisure PA | Walk for leisure (METs*min*days) + Moderate PA for leisure (METs*min*days) + Vigorous PA for leisure (METs*min*days) |
| Total PA | Walking MET minutes/week + Total Moderate MET minutes/week + Total Vigorous MET minutes/week. |

Table S3. Sensitivity analysis to models including country for associations between community population density and population density change and individual-level physical activity.

| **Adjusted Model** | | | | | **Adjusted Model** | | | |
| --- | --- | --- | --- | --- | --- | --- | --- | --- |
|  | **All** | **HIC** | **MIC** | **LIC** | **All** | **HIC** | **MIC** | **LIC** |
| **Population Density at baseline (β per IQR = 4428 people/sq. km)** | | | | | **5-year change rate in population density (β per IQR = 2% increase per year)** | | | |
| Total PA | -8.4  (-12.5, -4.2) | -16.23  (-25.8, -5.4) | -4.3  (-11.8, 3.8) | -12.4  (-19.3, -4.9) | -17.6  (-26.6, -7.6) | -24.5  (-37.6, -8.5) | -15.1  (-27.2, -1.3) | -15.2  (-34.6, 10.1) |
| Transport | 1.8  (-4.4, 8.0) | 100.4  (62.7, 146.8) | 29.3  (17.5, 42.2) | -16.2  (-21.4, -10.7) | 8.7  (-6.3, 26.4) | 5.5  (-24.3, 47.1) | 13.6  (-5.2, 36.3) | -15.9  (-36.7, 11.6) |
| Recreation | 9.3  (3.3, 15.5) | 5.9  (-12.5,28.2) | 38.2  925.5, 52.0) | -8.5  (-13.3, -3.40 | 5.2  (-8.4, 20.9) | 30.8  (-3.5, 77) | 8.7  (-8.9, 29.7) | -22.4  (-38.6, -1.9) |
| Household | -12.4  (-16.7, -7.9) | -38.6  (-47.4, -28.2) | -16.2  (-23.2, -8.6) | -8.4  (-13.7,-2.8) | -25.6  (-34.5, -15.5) | -35.2  (-49.3, -17.3) | -24.1  (-35.4, -10.8) | -13.8  (-35.6, 15.3) |
| Occupation | -16.2  (-21.4, -10.7) | -23  (-37.7, -5.6) | -29.8  (-37.3, -21.4) | -8.5  (-14.2, -2.3) | -20.4  (-32.0, -6.8) | -38.8  (-53.6, -19.2) | -17.7  (-33.3, 1.5) | -0.3  (-25.7, 33.6) |
| Age, sex, baseline year, education, wealth index, country income level, chronic disease at baseline, BMI, and nested random intercept for each community by center.  # β coefficients represent percent change in MET minutes per week of total or domain specific physical activity. | | | | | | | | |

Table S4. Sensitivity analysis to models including country for associations between community baseline impervious surface area and impervious surface area change and individual-level physical activity.

| **Adjusted Model** | | | | | **Adjusted Model** | | | |
| --- | --- | --- | --- | --- | --- | --- | --- | --- |
|  | **All** | **HIC** | **MIC** | **LIC** | **All** | **HIC** | **MIC** | **LIC** |
| **Impervious Area at baseline (β per IQR = 73%)** | | | | | **5-year change rate in impervious area (log) (β per IQR = 2 % increase)** | | | |
| Total PA | -24.5  (-33.9, -13.7) | -21.2  (-33.4, -6.7) | -16.1  (-29.1, -0.7) | -60.5  (-72.9, -42.4) | 3.9  (-5.9, 14.7) | -2.9  (-12.8, 7.9) | 4.2  (-9.4, 20.0) | 11.7  (-22.4, 60.8) |
| Transport | 68.6  (42.2, 99.9) | 137.8  (76.2, 220) | 105.2  (67.8, 150.9) | -71.8  (-81.4, -57.2) | -0.5  (-12.7, 13.3) | -8.4  (-23.7, 10.1) | 4.8  (-12.6, 25.7) | -7.5  (-34.4, 30.4) |
| Recreation | 58.5  (35.1, 86.1) | 11.7  (-14.4, 45.9) | 91.6  (56.7, 134.4) | -29.7  (-51.5,1.8) | -13.0  (-23.2, -1.5) | 1.1  (-15.1, 20.2) | -17.9  (-31.0, -2.3) | -9.5  (-30.9, 18.6) |
| Household | -40.3  (-48.3, -31.1) | -44.1  (-55.3, -30.1) | -38.7  (-48.7, -26.8) | -45.0  (-63.9, -16.2) | 9.0  (-2.8, 22.3) | 5.3  (-8.7, 21.6) | 10.0  9-6.3, 29.2) | 0.7  (-30.5, 45.9) |
| Occupation | -53.3  (-61.1, -44.1) | -37.7  (-53.3, -16.8) | -54.5  (-63.9, -42.5) | -38.2  (-59.9, -4.7) | 18.4  (3.4, 35.6) | 22.3  (0.7, 48.5) | 1.7  (-15.8, 23.1) | 21.7  (-14.1, 72.5) |
| Adjusted Model: Age, sex, baseline year, education, wealth index, country income level, chronic disease at baseline, BMI, and nested random intercept for each community by center.  # β coefficients represent percent change in MET minutes per week of total or domain specific physical activity. | | | | | | | | |

Table S5. Sensitivity analysis to models including joint model including population density and impervious area for associations between community population density and population density change and individual-level physical activity.

| **Adjusted Model** | | | | | **Adjusted Model** | | | |
| --- | --- | --- | --- | --- | --- | --- | --- | --- |
|  | **All** | **HIC** | **MIC** | **LIC** | **All** | **HIC** | **MIC** | **LIC** |
| **Population Density at baseline (β per IQR = 4428 people/sq. km)** | | | | | **5-year change rate in population density (β per IQR = 2% increase per year)** | | | |
| Total PA | -8.4  (-12.9, -3.7) | -8.4  (-23.1, 8.8) | 1.6  (-7.5, 11.8) | -12.4  (-19.0, -5.3) | -8.3  (-19.3, 4.2) | -42.6  (-50.1, -34.0) | -4.1  (-19.3, 13.9) | -3.5  (-31.1, 35.0) |
| Transport | -12.4  (-17.9, -6.5) | 60.1  (20.6, 113.8) | 8.2  (-3.2, 21.2) | -8.4  (-16.1, -0.1) | 19.1  (0.6, 41.06) | -14.2  (-35.2, 13.6) | 18.6  (-4.7, 47.8) | 5.7  (-26.9, 52.9) |
| Recreation | 0.7  (-5.4, 7.1) | -1.2  (-24.5, 29.2) | 24.7  (11.4, 39.6) | -8.4  (-14.5, -2.0) | 12.8  (93.6, 31.9) | 26.4  (-7.3, 72.6) | 11.3  (-10.1, 37.3) | -0.9  (-23.5, 28.5) |
| Household | -8.5  (-13.2, -3.4) | -29.8  (-43.5, -12.8) | -1.2  (-10.9, 9.6) | -12.4  (-19.1, -5.2) | -15.15  (-26.7, -1.7) | -50.5  (-59.2, -40.2) | -10.7  (-26.6, 8.6) | -10.4  (-36.5, 26.3) |
| Occupation | -8.5  (-14.7, -1.8) | -8.4  (-31.2, 21.8) | -16.2  (-27.1, -3.7) | -4.3  (-12.2, 4.3) | -24.6  (-36.4, -10.6) | -39.4  (-53.5, -20.8) | -21.9  (-38.1, -1.6) | 8.9  (-21.8, 51.8) |
| Adjusted Model: Age, sex, baseline year, education, wealth index, country income level, chronic disease at baseline, BMI, and nested random intercept for each community by center.  # β coefficients represent percent change in MET minutes per week of total or domain specific physical activity. | | | | | | | | |

Table S6. Sensitivity analysis to models including joint model including population density and impervious area for associations between community baseline impervious surface area and impervious surface area change and individual-level physical activity.

| **Adjusted Model** | | | | | **Adjusted Model** | | | |
| --- | --- | --- | --- | --- | --- | --- | --- | --- |
|  | **All** | **HIC** | **MIC** | **LIC** | **All** | **HIC** | **MIC** | **LIC** |
| **Impervious Area at baseline (β per IQR = 73%)** | | | | | **5-year change rate in impervious area (log) (β per 2 % increase over 5 years)** | | | |
| Total PA | -17.8  (-29.2, -4.5) | -12.6  (-31.2, 10.8) | -23.4  (-36.9, -6.8) | -32.9  (-60.8, 14.6) | 2.3  (-7.0, 12.6) | -0.9  (-10.4, 9.5) | 1.6  (-11.2, 16.1) | 7.1  (-22.6, 48.1) |
| Transport | 107.5  (70.7, 152.1) | 60.9  (9.5, 136.4) | 83.4  (44.5, 132.5) | -52.5  (-74.1, -13.1) | -2.3  (-13.8, 10.8) | -8.6  (-23.7, 9.7) | 1.4  (-14.6, 20.6) | -10.3  (-36.7, 27.1) |
| Recreation | 53.2  (27.3, 84.5) | 16.7  (-18.9, 68.2) | 37.3  (7.8, 74.9) | 22.2  (-22.7, 93.3) | -11.6  (-21.7, -0.2) | 1.5  (-14.5, 20.4) | -17.5  (-30.5, -2.01) | -7.3  (-27.8, 18.9) |
| Household | -38.6  (-47.8, -27.9) | -22.0  (-42.2, 5.2) | -42.6  (-53.5, -29.2) | -22.6  (-55.1, 33.1) | 8.5  (-2.9, 21.3) | 6.9  (-7.1, 23.2) | 6.9  (-8.3, 24.7) | 5.4  (-24.9, 48.1) |
| Occupation | -43.2  (-54.0, -29.9) | -28.4  (-51.6, 6.1) | -40.7  (-55.2, -21.4) | -12.1  (-51.7, 60.1) | 15.2  (0.9, 31.4) | 22.4  (1.3, 47.7) | -3.7  (-19.9, 15.7) | 16.9  (-17.5, 65.7) |
| Adjusted Model: Age, sex, baseline year, education, wealth index, country income level, chronic disease at baseline, BMI, and nested random intercept for each community by center.  # β coefficients represent percent change in MET minutes per week of total or domain specific physical activity. | | | | | | | | |

Table S7. Sensitivity analysis to models using un-transformed PA outcomes for associations between community population density and population density change and individual-level physical activity

| **Adjusted Model** | | | | | **Adjusted Model** | | | |
| --- | --- | --- | --- | --- | --- | --- | --- | --- |
|  | **All** | **HIC** | **MIC** | **LIC** | **All** | **HIC** | **MIC** | **LIC** |
| **Population Density at baseline (β per IQR = 4428 people/sq. km)** | | | | | **5-year change rate in population density (β per IQR = 2% increase per year)** | | | |
| Total PA | -275  (-367, -184) | -503  (-797, -209) | -401  (-570, -231) | -168  (-251, -85) | -353  (-584, -123) | 1098  (890, 1307) | -175  (-490, 139) | -310  (-688, 66) |
| Transport | 9  (-10, 28) | 142  (89, 195) | 60  (24, 97) | -28  (-41, -14) | 30  (-19, 80) | -92  (-136, -49) | 65  (-4, 133) | -32  (-84, 20) |
| Recreation | 31  (13, 47) | 61  (-20, 142) | 81  (51, 110) | 5  (-6, 16) | 46  (3, 89) | -66  (-128, -3) | 61  (6, 116) | 28  (-23, 80) |
| Household | -121  (-159, -82) | -482  (-647, -316) | -184  (-253, -114) | -70  (-109, -31) | -184  (-283, -84) | -491  (-623, -358) | -139  (-269, -10) | -115  (-269, 38) |
| Occupation | -189  (-258, -120) | -193  (-415, 28) | -357  (-485, -229) | -62  (-126, 0.5) | -250  (-421, -79) | 455  (294, 616) | -182  (-420, 55) | -169  (-461, 122) |
| Adjusted Model: Age, sex, baseline year, education, wealth index, country income level, chronic disease at baseline, BMI, and nested random intercept for each community by center.  # β coefficients represent percent change in MET minutes per week of total or domain specific physical activity. | | | | | | | | |

Table S8. Sensitivity analysis to models using un-transformed PA outcomes for associations between community baseline impervious surface area and impervious surface area change and individual-level physical activity.

| **Adjusted Model** | | | | | **Adjusted Model** | | | |
| --- | --- | --- | --- | --- | --- | --- | --- | --- |
|  | **All** | **HIC** | **MIC** | **LIC** | **All** | **HIC** | **MIC** | **LIC** |
| **Impervious Area at baseline (β per IQR = 73%)** | | | | | **5-year change rate in impervious area (log) (β per 2 % increase over 5 years)** | | | |
| Total PA | -1183  (-1448, -919) | -975  (-1356, -593) | -1179  (-1520, -837) | -1084  (-1639, -530) | 11  (-174,196) | 59  (-191, 308) | -14  (-277, 250) | 195  (-203, 594) |
| Transport | 90  (32, 148) | 169  (94, 245) | 128  (52, 205) | -172  (-257, -88) | -6  (-53, 41) | -14  (-64, 35) | 21  (-49, 92) | -38  (-94, 19) |
| Recreation | 157  (107, 207) | 70  (-40, 180) | 195  (133, 258) | -3  (-74, 69) | -25  (-66,16) | 41  (-30, 112) | -34  (-89, 21) | -6  (-63, 50) |
| Household | -587  (-696, -477) | -666  (-887, -445) | -595  (-732, -458) | -494  (-739, -250) | -48  (-135, 38) | -6  (-146, 132) | -21  (-143, 100) | 9  (-129, 148) |
| Occupation | -830  (-1030, -630) | -467  (-760, -173) | -896  (-1157, -635) | -431  (-856, -6) | 68  (-64, 200) | 111  (-77, 299) | -5  (-190, 180) | 186  (-173, 550) |
| Adjusted Model: Age, sex, baseline year, nested random intercept for each community by center, education, wealth index, country income level, chronic disease at baseline, BMI, baseline population density or baseline impervious area  # β coefficients represent percent change in MET minutes per week of total or domain specific physical activity. | | | | | | | | |

**Funding Support, PURE Investigators and Primary Country Based Institutions**

**Funding/Support:**

Dr S Yusuf is supported by the Mary W Burke endowed chair of the Heart and Stroke Foundation of Ontario.

The PURE study is an investigator-initiated study that is funded by the Population Health Research Institute, Hamilton Health Sciences Research Institute (HHSRI), the Canadian Institutes of Health Research, Heart and Stroke Foundation of Ontario, Support from Canadian Institutes of Health Research’s Strategy for Patient Oriented Research, through the Ontario SPOR Support Unit, as well as the Ontario Ministry of Health and Long-Term Care and through unrestricted grants from several pharmaceutical companies [with major contributions from AstraZeneca (Canada), Sanofi-Aventis (France and Canada), Boehringer Ingelheim (Germany and Canada), Servier, and GlaxoSmithKline], and additional contributions from Novartis and King Pharma and from various national or local organisations in participating countries.

These include: **Argentina:** Fundacion ECLA **(Estudios Clínicos Latino America)** ; **Bangladesh**: Independent University, Bangladesh and Mitra and Associates; **Brazil:** Unilever Health Institute, Brazil; **Canada:** This study was supported by an unrestricted grant from Dairy Farmers of Canada and the National Dairy Council (U.S.), Public Health Agency of Canada and Champlain Cardiovascular Disease Prevention Network; **Chile:** Universidad de La Frontera [DI13-PE11]; **China:** National Center for Cardiovascular Diseases and ThinkTank Research Center for Health Development; **Colombia:** Colciencias (grant 6566-04-18062 and grant 6517-777-58228); **India:** Indian Council of Medical Research; **Malaysia:** Ministry of Science, Technology and Innovation of Malaysia (grant number: 100-IRDC/BIOTEK 16/6/21 [13/2007], and 07-05-IFN-BPH 010), Ministry of Higher Education of Malaysia (grant number: 600-RMI/LRGS/5/3 [2/2011]), Universiti Teknologi MARA, Universiti Kebangsaan Malaysia (UKM-Hejim-Komuniti-15-2010); **occupied Palestinian territory:** the United Nations Relief and Works Agency for Palestine Refugees in the Near East, occupied Palestinian territory; International Development Research Centre, Canada; **Philippines:** Philippine Council for Health Research and Development; **Poland:** Polish Ministry of Science and Higher Education (grant number: 290/W-PURE/2008/0), Wroclaw Medical University; **Saudi Arabia:** Saudi Heart Association, Saudi Gastroenterology Association, Dr.Mohammad Alfagih Hospital, The Deanship of Scientific Research at King Saud University, Riyadh, Saudi Arabia (Research group number: RG -1436-013); **South Africa:** The North-West University, SA and Netherlands Programme for Alternative Development, National Research Foundation, Medical Research Council of South Africa, The South Africa Sugar Association, Faculty of Community and Health Sciences; **Sweden:** Grants from the Swedish state under the Agreement concerning research and education of doctors; the Swedish Heart and Lung Foundation; the Swedish Research Council; the Swedish Council for Health, Working Life and Welfare, King Gustaf V:s and Queen Victoria Freemason’s Foundation, AFA Insurance; **Turkey:** Metabolic Syndrome Society, AstraZeneca, Sanofi Aventis; **United Arab Emirates:** Sheikh Hamdan Bin Rashid Al Maktoum Award For Medical Sciences and Dubai Health Authority, Dubai.

**Role of Sponsor:** The external funders and sponsors had no role in the design and conduct of the study; in the collection, analysis, and interpretation of the data; in the preparation, review, or approval of the

manuscript; or in the decision to submit the manuscript for publication.

**PURE Project Office Staff, National Coordinators, Investigators, and Key Staff:**

**Project office (Population Health Research Institute, Hamilton Health Sciences and McMaster University, Hamilton, Canada):** S Yusuf* (Principal Investigator).

S Rangarajan (Program Manager); K K Teo, S S Anand, C K Chow, M O’Donnell, A Mente, D Leong, A Smyth, P Joseph, M Duong, O Kurmi, R D’Souza, M Walli-Attaei, B Balaji, R Naito, S Islam (Statistician), W Hu (Statistician), C Ramasundarahettige (Statistician), P Sheridan (Statistician), S Bangdiwala, L Dyal, M Dehghan (Nutrition Epidemiologist), A Aliberti, A Reyes, A Zaki, B Connolly, B Zhang, D Agapay, D Krol, E McNeice, E Ramezani, F Shifaly, G McAlpine, I Kay, J Rimac, J Swallow, M Di Marino, M Jakymyshyn, M(a) Mushtaha, M(o) Mushtaha, M Trottier, N Aoucheva, N Kandy, P Mackie, R Buthool, R Patel, R Solano, S Gopal, S Ramacham, S Trottier

**Core Laboratories**: G Pare, M McQueen, S Lamers, J Keys (Hamilton), X Wang (Beijing, China), A Devanath (Bangalore, India).

**Argentina:** R Diaz*, A Orlandini, P Lamelas, M L Diaz, A Pascual, M Salvador, C Chacon; **Bangladesh:** O Rahman*, R Yusuf*, S A K S. Ahmed, T Choudhury, M Sintaha, A Khan, O Alam, N, Nayeem, S N Mitra, S Islam, F Pasha; **Brazil:** A Avezum*, C S Marcilio, A C Mattos, G B Oliveira; **Canada:**  K Teo***,** S Yusuf*****, Sumathy Rangarajan, A Arshad, B Bideri, I Kay, J Rimac, R Buthool, S Trottier, G Dagenais, P Poirier, G Turbide, AS Bourlaud, A LeBlanc De Bluts, M Cayer, I Tardif, M Pettigrew, S Lear, V de Jong, A N Saidy, V Kandola, E Corber, I Vukmirovich, D Gasevic, A Wielgosz, A Pipe, A Lefebvre, A Pepe, A Auclair, A Prémont, A S Bourlaud; **Chile:** F Lanas*, P Serón, M J Oliveros, F Cazor, Y Palacios; **China:** Li Wei*, Liu Lisheng*, Bo Jian, Hu Bo, Yin Lu, Zhao Wenhua, Zhang Hongye, Jia Xuan, Sun Yi, Wang Xingyu, Zhao Xiuwen, He Xinye, Chen Tao, Chen Hui, Chang Xiaohong, Deng Qing, Cheng Xiaoru, Deng Qing, Xie Liya, Liu Zhiguang, Li Juan, Li Jian, Liu Xu, Ren Bing, Sun Yi, Wang Wei, Wang Yang, Yang Jun, Zhai Yi, Zhang Hongye, Zhao Xiuwen,Zhu Manlu, Lu Fanghong, Wu Jianfang, Li Yindong, Hou Yan, Zhang Liangqing, Guo Baoxia, Liao Xiaoyang, Zhang Shiying, BianRongwen, TianXiuzhen, Li Dong, Chen Di, Wu Jianguo, Xiao Yize, Liu Tianlu, Zhang Peng, Dong Changlin, Li Ning, Ma Xiaolan, Yang Yuqing, Lei Rensheng, Fu Minfan, He Jing, Liu Yu, Xing Xiaojie, Zhou Qiang; **Colombia:** P Lopez-Jaramillo*, P A Camacho-Lopez, M Perez, J Otero-Wandurraga, D I Molina, C Cure-Cure, JL Accini, E Hernandez, E Arcos, C Narvaez, A Sotomayor, F Manzur, H Garcia, G Sanchez, F Cotes, A Rico, M Duran, C Torres; **India: Bangalore -** P Mony *, M Vaz*, S Swaminathan, AV Bharathi, K Shankar, A V Kurpad, K G Jayachitra, H A L Hospital, AR Raju, S Niramala, V Hemalatha, K Murali, C Balaji, A Janaki, K Amaranadh, P Vijayalakshmi, **Chennai** - V Mohan*, R M Anjana, M Deepa, K Parthiban, L Dhanasekaran, SK Sundaram, M Rajalakshmi, P Rajaneesh, K Munusamy, M Anitha, S Hemavathy, T Rahulashankiruthiyayan, D Anitha, R. Dhanasekar, S. Sureshkumar, D Anitha, K Sridevi, **Jaipur** - R Gupta, R B Panwar, I Mohan, P Rastogi, S Rastogi, R Bhargava, M Sharma, D Sharma, **Trivandrum** - V Raman Kutty, K Vijayakumar, V Ambili, Arunlal AR Nair, K Ajayan, G Rajasree, AR Renjini, A Deepu, B Sandhya, S Asha, H S Soumya, **Chandigarh**- R Kumar, M Kaur, P V M Lakshmi, V Sagar J S Thakur, B Patro, R Mahajan, A Josh, G Singh, K Sharma, P Chaudary, **Iran:** R Kelishadi*, A Bahonar, N Mohammadifard, H Heidari, **Kazakhstan:** K Davletov*, B Assembekov, B Amirov; **Kyrgyzstan:** E Mirrakhimov*, S Abilova, U Zakirov, U Toktomamatov; **Malaysia: UiTM -** K Yusoff*, T S Ismail, K Ng, A Devi, N Mat-Nasir, AS Ramli, MNK Nor-Ashikin, R Dasiman, MY Mazaouspavina, F Ariffin, M Miskan, H Abul-Hamid, S Abdul-Razak, N Baharudin, NMN Mohd-Nasir, SF Badlishah-Sham, M Kaur, M Koshy, F A Majid, N A Bakar, N Zainon, R Salleh, SR Norlizan, NM Ghazali, M Baharom, H Zulkifli, R Razali, S Ali, CWJCW Hafar, F Basir; **UKM** - Noorhassim Ismail, M J Hasni, M T Azmi, M I Zaleha, R Ismail, K Y Hazdi, N Saian, A Jusoh, N Nasir, A Ayub, N Mohamed, A Jamaludin, Z Rahim; **Occupied Palestinian Territory:** R Khatib*, U Khammash, R Giacaman; **Pakistan:** R Iqbal*, R Khawaja, I Azam, K Kazmi; **Peru:** J Miranda*, A Bernabe Ortiz, W Checkley, R H Gilman, L Smeeth, R M Carrillo, M de los Angeles, C Tarazona Meza**;** **Philippines:** A Dans*, H U Co, J T Sanchez, L Pudol, C Zamora-Pudol, L A M Palileo-Villanueva, M R Aquino, C Abaquin, SL Pudol, K Manguiat, S Malayang; **Poland:** W Zatonski*, A Szuba, K Zatonska, R Ilow**^#^**, M Ferus, B Regulska-Ilow, D Różańska, M Wolyniec; **Saudi Arabia:** KF AlHabib*, M Alshamiri, HB Altaradi, O Alnobani, N Alkamel, M Ali, M Abdulrahman, R Nouri; **South Africa:** L Kruger^*^, A Kruger^#^, P Bestra, H Voster, A E Schutte, E Wentzel-Viljoen, FC Eloff, H de Ridder, H Moss, J Potgieter, A Roux, M Watson, G de Wet, A Olckers, J C Jerling, M Pieters, T Hoekstra, T Puoane, R Swart*, E Igumbor, L Tsolekile, K Ndayi, D Sanders, P Naidoo, N Steyn, N Peer, B Mayosi^#^, B Rayner, V Lambert, N Levitt, T Kolbe-Alexander, L Ntyintyane, G Hughes, J Fourie, M Muzigaba, S Xapa, N Gobile , K Ndayi, B Jwili, K Ndibaza, B Egbujie; **Sweden** A Rosengren*, K Bengtsson Boström, A Rawshani, A Gustavsson, M Andreasson, L Wirdemann; **Tanzania:** K Yeates*, M Oresto, N West **Turkey:** A Oguz*, N Imeryuz, Y Altuntas, S Gulec, A Temizhan, K Karsidag, K B T Calik, A K Akalin, O T Caklili, M V Keskinler, K Yildiz; **United Arab Emirates:** A H Yusufali, F Hussain, M H S Abdelmotagali, D F Youssef, O Z S Ahmad, F H M Hashem, T M Mamdouh, F M AbdRabbou, S H Ahmed, M A AlOmairi, H M Swidan, M Omran, N A Monsef ; **Zimbabwe:** J Chifamba*, T Ncube, B Ncube, C Chimhete, G K Neya, T Manenji, L Gwaunza, V Mapara, G Terera, C Mahachi, P Murambiwa, R Mapanga, A Chinhara

*National Coordinator

^#^ Deceased

**PURE Country Institution Names:**

|  | **Institution** |
| --- | --- |
| **South Africa** | Faculty of Health Science  North-West University  Potchefstroom Campus |
|  | University of the Western Cape  Department of Dietetics and Nutrition  Private Bag X17, 7535  Bellville, South Africa |
| **Zimbabwe** | University of Zimbabwe  College of Health Sciences  Physiology Department  Harare, Zimbabwe |
| **Tanzania** | Pamoja Tunaweza Women Center, Moshi, Tanzania  Division of Nephrology, Department of Medicine  Queen's University |
| **China** | National Centre for Cardiovascular Diseases  Cardiovascular Institute & Fuwai Hospital  Chinese Academy of Medical Sciences  167, Bei Li Shi Lu, Beijing, China |
|  | Fuwai Hospital  167 Beilishi Rd. Xicheng District  Beijing. 100037 China |
| **Philippines** | University of Philippines, Section of Adult Medicine & Medical Research Unit, Manila, Philippines |
| **Pakistan** | Department of Community Health Sciences and Medicine  Aga Khan University  Stadium Road, P.O Box 3500  Karachi Pakistan |
| **India, Bangalore** | St John's Medical College and Research Institute Bangalore 560034, India |
| **India, Chennai** | Madras Diabetes Research Foundation &  Dr. Mohan’s Diabetes Specialities Centre, Chennai |
| **India Jaipur** | Eternal Heart Care Centre and Research Institute, Jaipur |
| **India, Trivandrum** | Health Action by People,  Thiruvananthapuram, Kerala, 695011 INDIA |
| **India, Chandigarh** | School of Public Health, Post Graduate Institute of Medical Education & Research, Chandigarh (India) |
| **Bangladesh** | Independent University, Bangladesh  Bashundhara, Dhaka  Bangladesh |
| **Malaysia** | Universiti Teknologi MARA, Sungai Buloh, Selangor, Malaysia AND UCSI University, Cheras, Selangor, Malaysia |
|  | Department of Community Health. Faculty of Medicine. University Kebangsaan Malaysia. Kuala Lumpur. Malaysia |
| **Poland** | Wroclaw Medical University Department of Internal Medicine; Department of Social Medicine Borowska 213 street; 50- 556 Wroclaw, Poland |
|  | Department of Epidemiology,  The Maria Skłodowska-Curie Memorial Cancer Center and Institute of Oncology  02-034 Warsaw, 15B Wawelska str.  Poland |
| **Turkey** | Istanbul Medeniyet University  Istanbul, Turkey |
| **Sweden** | Sahlgrenska Academy  University of Gothenburg  Sweden |
| **Iran** | Isfahan Cardiovascular Research Center, Isfahan Research Institute  Isfahan University of Medical Sciences, Isfahan, Iran |
| **UAE** | Dubai Medical University, Hatta Hospital, Dubai Health Authority, Dubai, United Arab Emirates |
| **Saudi Arabia** | Department of Cardiac Sciences, King Fahad Cardiac Center  College of Medicine  King Saud University  Riyadh, Saudi Arabia |
| **Palestine** | Institute of Community and Public Health, Birzeit University, Ramallah, occupied Palestinian territory |
| **Canada** | Université Laval Institut universitaire de cardiologie et de pneumologie de Québec, Quebec  Canada G1V 4G5 |
|  | Simon Fraser University,  Dept. of Biomedical Physiology & Kinesiology, BC, Canada |
|  | Department of Medicine,  University of Ottawa,  Ottawa, Canada |
|  | Population Health Research Institute, McMaster University, Hamilton Health Sciences, Hamilton, Ontario, Canada |
| **Argentina** | Estudios Clinicos Latinoamerica ECLA  Rosario, Santa Fe  Argentina  Department of Chronic Diseases  South American Center of Excellence for Cardiovascular Health (CESCAS)  Institute for Clinical Effectiveness and Health Policy (IECS) |
| **Brazil** | Dante Pazzanese Institute of Cardiology;  Hospital Alemao Oswaldo Cruz  Sao Paulo, SP Brazil |
| **Colombia** | Facultad de Ciencias de la Salud, Universidad de Santander (UDES), Bucaramanga, Santander,  Fundacion Oftalmologica de Santander (FOSCAL)  Floridablanca-Santander, Colombia |
| **Chile** | Universidad de La Frontera  Temuco, Chile |
| **Ecuador** | DECANO  Facultad de Ciencias de la Salud Eugenio Espejo  Universidad Tecnológica Equinoccial  Dirección: Av. Mariscal Sucre s/n y Av. Mariana de Jesús, Quito Ecuador |
| **Peru** | CRONICAS Centro de Excelencia en Enfermedades Crónicas \| [www.cronicas-upch.pe](http://www.cronicas-upch.pe)  Universidad Peruana Cayetano Heredia \| www.upch.edu.pe  Av. Armendáriz 497, Miraflores, Lima |
| **Russia** | Research Institute for Complex Issues of Cardiovascular Diseases, Kemerovo, Russia  Institute For Medical Education, Yaroslav-the-Wise Novgorod State University Ministry of Education and Science of the Russian Federation  Russia, Saint-Petersburg, 197022,  Karpovka river emb., Bld.13, office 28 |
| **Kazakhstan** | Research Institute of Cardiology & Internal Diseases, Almaty, Kazakhstan |
| **Kyrgyzstan** | Kyrgyz Society of Cardiology, National Center of Cardiology and Internal Disease, Bishkek, Kyrgyzstan |
